# Reversible Microscale Assembly of Nanoparticles Driven by the Phase Transition of a Thermotropic Liquid Crystal

**DOI:** 10.1021/acsnano.2c09203

**Published:** 2023-05-24

**Authors:** Niamh Mac Fhionnlaoich, Stephen Schrettl, Nicholas B. Tito, Ye Yang, Malavika Nair, Luis A. Serrano, Kellen Harkness, Paulo Jacob Silva, Holger Frauenrath, Francesca Serra, W. Craig Carter, Francesco Stellacci, Stefan Guldin

**Affiliations:** † Department of Chemical Engineering, University College London, London WC1E 7JE, United Kingdom; ‡ Institute of Materials, École Polytechnique Fédérale de Lausanne, 1015 Lausanne, Switzerland; ¶ Department of Applied Physics and Science Education, Eindhoven University of Technology, 5612 AP Eindhoven, The Netherlands; § Department of Physics, Chemistry and Pharmacy, University of Southern Denmark, 5230 Odense, Denmark; ∥ Department of Materials Science and Engineering, Massachusetts Institute of Technology, Cambridge, Massachusetts 02139, United States

**Keywords:** hierarchical, liquid crystals, nanoparticles, phase transition, self-assembly, soft matter

## Abstract

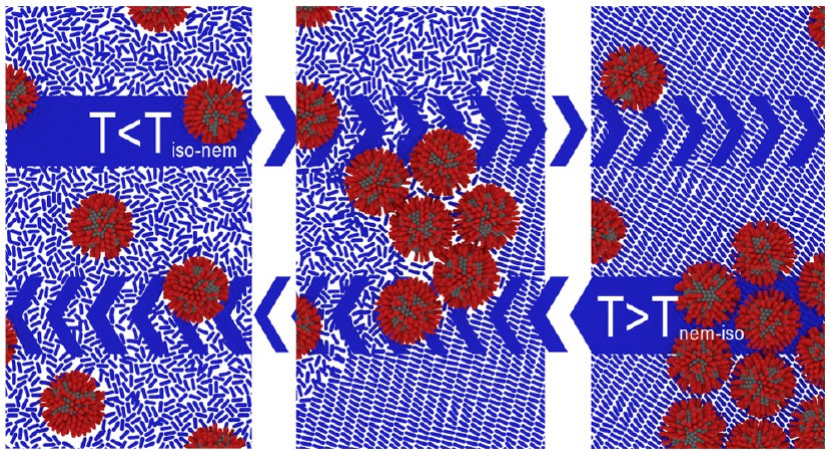

The arrangement of nanoscale building blocks into patterns with microscale periodicity is challenging to achieve via self-assembly processes. Here, we report on the phase-transition-driven collective assembly of gold nanoparticles in a thermotropic liquid crystal. A temperature-induced transition from the isotropic to the nematic phase under anchoring-driven planar alignment leads to the assembly of individual nanometer-sized particles into arrays of micrometer-sized agglomerates, whose size and characteristic spacing can be tuned by varying the cooling rate. Phase field simulations coupling the conserved and nonconserved order parameters exhibit a similar evolution of the morphology as the experimental observations. This fully reversible process offers control over structural order on the microscopic level and is an interesting model system for the programmable and reconfigurable patterning of nanocomposites with access to micrometer-sized periodicities.

## Introduction

Apart from their widespread use in display technologies, liquid crystals (LCs) have attracted considerable interest in various fields of soft matter research.^1−3^ LCs offer intriguing opportunities for colloid science.^4−6^ The elastic distortion of the mesogen director field around colloidal inclusions, i.e., topological defects, enables colloidal assembly into organized patterns by long-range interactions.^7−11^ The interplay of anisotropic elastic and weakly screened electrostatic interactions offers orientational and positional order.^12^ Colloid-induced topological defects can act as templates for self-assembly processes on the molecular scale.^13^ Such LC-mediated colloidal interactions are size-dependent.^14,15^ Accordingly, the elastic interaction with the director field should vanish when the diameter of the colloidal particles is below the surface extrapolation length, λ = *K*/*W*, where *K* is the elastic constant of the nematic LC and *W* is the surface anchoring strength, a measure of the interaction between the particle surface and the mesogen.^16^ Depending on the type of mesogens and the nature of the colloidal surface, this typically results in a minimum size of around 10–100 nm below which no elastic distortion of the director field is expected.

Composites of LCs with nanoparticles (NPs) that are below this length scale are nevertheless promising candidates for novel optoelectronic applications, such as optical filters, metamaterials, polarizers, or switches.^17−24^ The large birefringence combined with versatile driving methods for switching make LC-NP composites an attractive material system for reconfigurable active plasmonic devices.^25^ This was recently experimentally demonstrated for Ag and Au NPs, where structural changes of the LC-NP composite were induced by a thermal stimulus and led to a tunable plasmonic response.^26,27^ A number of studies report on the successful coassembly of NPs by LCs into geometries that mimic the mesogen arrangement.^28−33^

Solubilizing such nanometer-sized objects in an LC is, however, a known and widespread challenge.^34^ The dense periodic packing of LC mesogens frustrates mixing and free diffusion of additives.^35^ To date, the most successful strategies to solubilize NPs rely on creating a ligand shell that resembles the mesogen environment, which is typically achieved by a mixture of mesogen-like ligands and shorter ligands that serve as spacers and thus create pockets for mesogen penetration.^36,37^ This approach enabled the preparation of LC-NP composites with NP loadings exceeding 50 wt %.^38^ At significant NP loadings, the presence of non-nematogenic species was found to have profound effects on the phase behavior of nematic LCs, and several theoretical and experimental studies have established phase diagrams of LC-NP composites.^38−40^

In principle, exploitation of liquid crystal phase transition processes offers access to structure formation beyond the nanometer length scale. This was first achieved via the cooling of a colloid-liquid crystal composite comprised of 0.5 μm-sized poly(methyl methacrylate) particles and the thermotropic LC 4′-pentyl-biphenyl-4-carbonitrile (5CB) below its isotropic-to-nematic transition.^41^ Several studies followed this approach to yield soft solids with high storage moduli, which can be related to the formation of a continuous network of particle aggregates trapped between nematic domains.^41−43^ Subsequent work on 1 μm-sized polystyrene microspheres suspended in a thermotropic liquid crystal mixture (E7) established that such network structures, often referred to as colloid-in-liquid crystal gels, may also be formed via two-step processes comprised of an initial spinodal decomposition of a colloidal dispersion in an isotropic phase of mesogens followed by the subsequent nucleation of nematic domains within the imposed spatial confinement.^44^ Recent studies focused on the structure formation behavior of nm- rather than μm-sized particles, aided by the above-described solubilization strategies with mesogen-resembling ligand shells. The presence of an isotropic–nematic coexistence region above a critical NP threshold enabled the formation of macroscopic NP-enriched networks.^45^ NP-based hollow microstructures were formed through a two-stage nematic nucleation process.^46,47^ Cooling of LC-NP composites also enabled the spatial separation of quantum dot clusters; however, control over the positional order was limited and only achieved by the introduction of macroscopic beads as nucleation points.^48,49^ While much progress has been made on colloid-liquid-crystal composites over the past 20 years, it remains a challenge for such and other self-assembling materials systems to spontaneously and reversibly arrange nanoscale building blocks into patterns of microscale periodicity with control over size and spatial arrangement.

In this work, we present how the temperature-induced isotropic-to-nematic phase transition of the thermotropic LC 4′-pentyl-biphenyl-4-carbonitrile (5CB) in an LC cell with planar alignment can drive a hierarchical assembly of nanometer-sized gold particles into micrometer-sized agglomerates. This process is not only reversible but offers control over the characteristic size and spacing of the resulting structures. We report on the dynamics of this process by experiments and accompanying phase-field simulations and correlate our findings to parameters such as the cooling rate, nanoparticle solubility, phase separation kinetics, and the properties of the nematic director field.

## Results and Discussion

Au NPs were made in a modular approach, which allowed independent control over both the NP size and ligand composition on the surface.^50^ First, oleylamine-protected NPs were synthesized and subsequently functionalized by ligand exchange with a 60:40 mol % mixture of 1-hexanethiol (HT) and 4′-(12-mercaptododecyloxy)biphenyl-4-carbonitrile (MDD-CBO). This led to an effective ligand composition on the NP surface of 61:39 ± 3 mol % according to analysis by NMR spectroscopy. The Au NP size distribution was 4.7 ± 0.7 nm according to TEM analysis. Another batch of Au NPs with a size distribution of 6.0 ± 1.0 nm and a comparable ligand composition yielded results similar to those reported here. The NPs were then transferred to 5CB in the isotropic phase at a concentration of 5  wt % following an established protocol using dichloromethane as a volatile solvent.^51^ Subsequently, the LC-NP composite was infiltrated into an LC cell with a defined gap thickness of 20  μm and homogeneous surface alignment. Further details on the NP synthesis, cleaning procedure, solubility, and material characterization can be found in the Supporting Information (Supporting Figures S3–S9). Interestingly, the NPs showed superior solubility in isotropic 5CB compared to various common solvents, including chloroform, chlorobenzene, and a 50/50 vol % mixture of acetonitrile and tetrahydrofuran (Supporting Figure S10).

**Figure 1:**
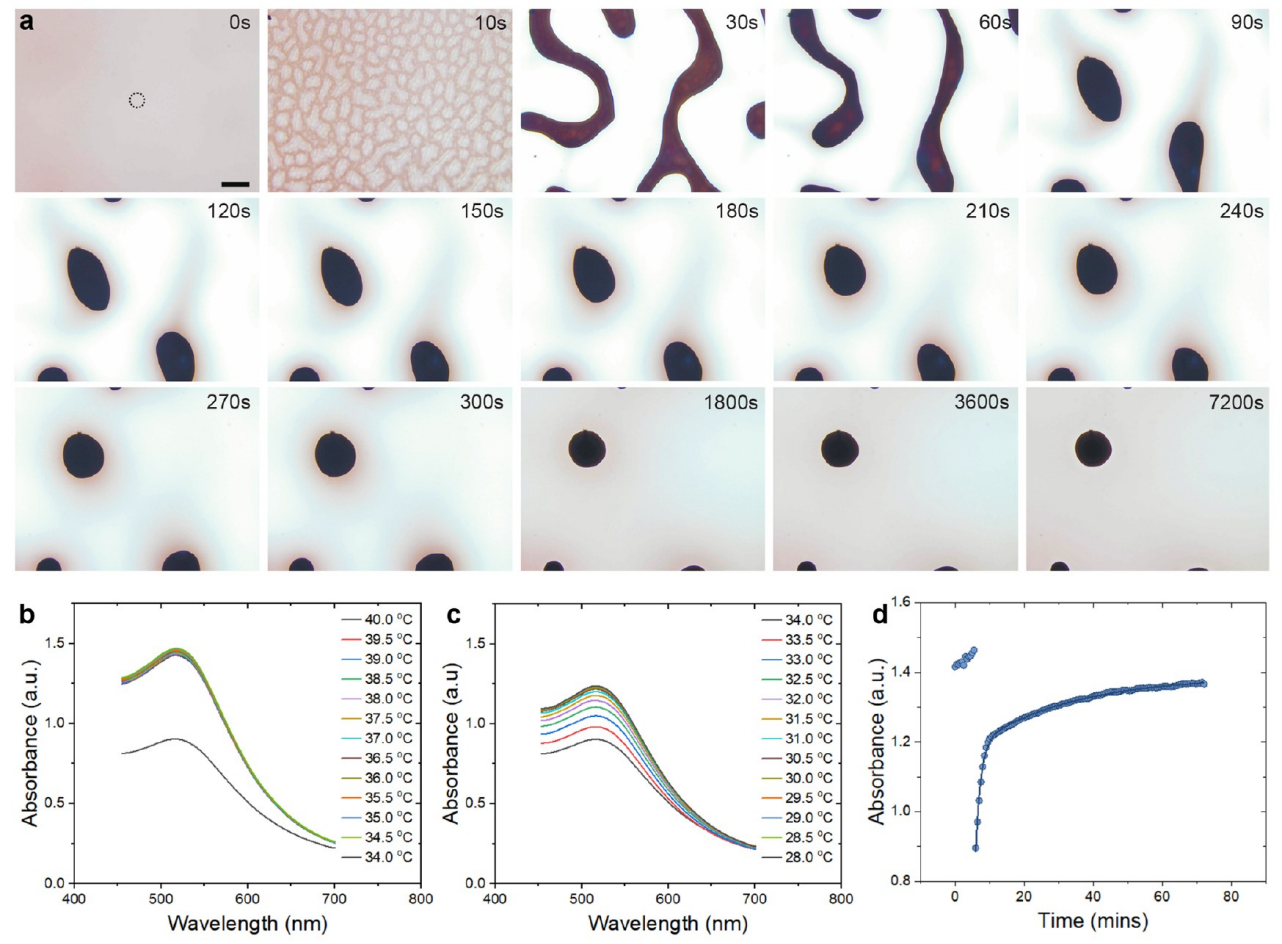
Liquid crystal phase transition and dynamics of nanoparticle diffusion into the nematic liquid crystal. (a) An example kinetic series of bright field microscopy images of the liquid crystal - nanoparticle composite upon cooling to 28.0 °C (1 °C min^–1^), with image acquisition commencing at 34.3 °C (20× objective). The scale bar represents 50  μm. (b–d) Absorbance microspectroscopy was carried out during this assembly process on a 25 μm-sized collection spot, represented by the dotted circle in (a). In situ absorbance spectra were recorded during cooling at a rate of 1 °C min^–1^ from 40.0 to 34.0 °C (b) and 34.0 to 28.0 °C (c). (d) Abs_max_ vs time *t*, where *t* = 0 corresponds to the start of the cooling process at 40.0 °C; the phase transition was initiated between 5.5 and 6 min and the terminal temperature of 28.0 °C was reached at 12 min and maintained for additional 60 min.

The dynamics of the LC phase transition and subsequent diffusion of NPs into the nematic phase are shown in Figure 1a. The LC-NP composite was initially heated at 50 °C for 2 h and subsequently cooled at a rate of 1 °C min^–1^ to 28 °C. In this experiment, the phase transition was observed to commence at 34.3 °C, which is in line with earlier studies that reported on the decrease of the phase transition temperature in the presence of NPs.^38,40,52,53^ A series of images was then taken at this temperature alongside microspectroscopic absorbance measurements by partial out-coupling of light. By analysis of the micrographs, the behavior observed upon cooling may be divided into two stages. In the initial stage within the first 60 s, a compartmentalization occurred into NP-depleted and NP-enriched regions that resembled a spinodal-type decomposition; over time, the characteristic continuous pattern evolved to yield isolated NP agglomerates at the end of the process. Cross-polarized optical microscopy revealed that during the phase transition process, the regions, which appeared brighter under bright field illumination in the initial decomposition phase, corresponded to nematic domains aligned with the homogeneous orientation of the cell, while the darker regions were nematic microdomains with random alignment (see Supporting Figures S11 and S12). As shown in the accompanying video of the isotropic–nematic transition under cross-polarization (see Supporting Video 1), the growth of the uniformly aligned nematic domains started shortly after the isotropic–nematic transition, once the randomly aligned domains formed at the transition became large enough to interact with the boundaries of the cell. We hypothesize that at the isotropic–nematic phase transition, the NPs tended to accumulate in the isotropic phase, as was observed in other systems.^48^ The subsequent growth of large, uniformly aligned domains, energetically favored by the cell alignment, promoted the segregation of NPs in the interstitial regions. Here, the NPs became more and more concentrated and, consequently, the receding phase became more and more disordered. Because of the high solubility of the NPs and the mesogenic nature of their ligands, the NP-enriched phase still retained some birefringence, which suggests a nonzero order parameter.

The findings of in situ absorbance microspectroscopy are shown in Figure 1b–d. As illustrated by the dotted circle in Figure 1a, a 25 μm-sized collection spot served for the acquisition of absorbance spectra. Since the absorbance of the 5CB mesogens within the 20 μm optical path was negligible in both the nematic and isotropic state, absorbance microspectroscopy provided a viable route to study the concentration of NPs in this assembly process with a μm-sized spatial resolution. Above the isotropic-to-nematic phase transition temperature, gradual cooling did not result in significant changes in the maximum absorbance of the LC-NP composite. However, once the phase transition was triggered at 34.3 °C, the maximum absorbance (Abs_max_) decreased drastically from 1.46 to 0.90 in the newly formed aligned nematic domains (Figure 1b), thus suggesting a fast partitioning of the NP in the less ordered regions. Absorbance spectra were subsequently acquired in 30 s intervals upon further cooling to the terminal temperature of 28.0 °C. As shown in Figure 1c and related microscopy images, a rapid increase in absorption to Abs_max_ = 1.22 within the first 5 min and a further increase to Abs_max_ = 1.37 was observed over time, which can be fitted by a logistic growth function (Figure 1d). Based on experiments at concentrations below 1.5 wt %, where no residual agglomerates formed, a similar molar attenuation coefficient was obtained for the LC-NP composite in the isotropic and nematic phase, respectively (Supporting Figure S13). The absorbance results are, thus, in line with a short-lived depletion of NPs in the planarly aligned nematic domains followed by subsequent diffusion of NPs from the enriched receding random nematic to the aligned nematic phase. After reaching steady state, the large majority of the NPs (93.8%) became dispersed in the aligned nematic phase with the residual fraction of NPs embedded as agglomerates in the composite matrix. Importantly, the shape of the spectral absorbance remained similar after the phase transition, i.e., apart from the ordered formation of μm-sized agglomerates, we did not observe the aggregation of individual AuNPs in the aligned nematic phase that would result in a spectral shift. We further emphasize on the role of the homogeneous substrate, which imposes an in-plane alignment of the mesogen director field. When cells without surface alignment were used, cooling of the LC-NP composite led to the formation of NP-enriched and NP-depleted regions but these remained in coexistence (see Supporting Figures S14 and S15), which is in line with earlier findings.^40,45^

To examine underlying effects, we developed a qualitative coarse-grained molecular dynamics model of a single NP complex with mixed ligand shell immersed in a host of nematogenic mesogens, which is discoursed in detail in the Supporting Information (Supporting Figures S1 and S2). We find that while interdigitation of the ligand shell generally facilitates solvation of the NP complex, the solvation free energy depends on whether the medium is isotropic or nematic. In the nematic phase, solvation of an object requires distorting the local order parameter, which introduces a free energy penalty to the system. A solvation barrier as driving force for a phase transition-driven collective assembly may be conceptualized as follows. Cooling of the LC-NP composite to the isotropic-to-nematic phase transition temperature *T*_iso–nem_ induces demixing and a short-term phase coexistence of NP-enriched regions with vanishing order parameter and NP-depleted aligned nematic regions. The buildup in NP concentration in the receding disordered phase then forces additional NPs to slowly diffuse into the aligned nematic phase until, eventually, micrometer-sized agglomerates of residual NPs are retained.

**Figure 2:**
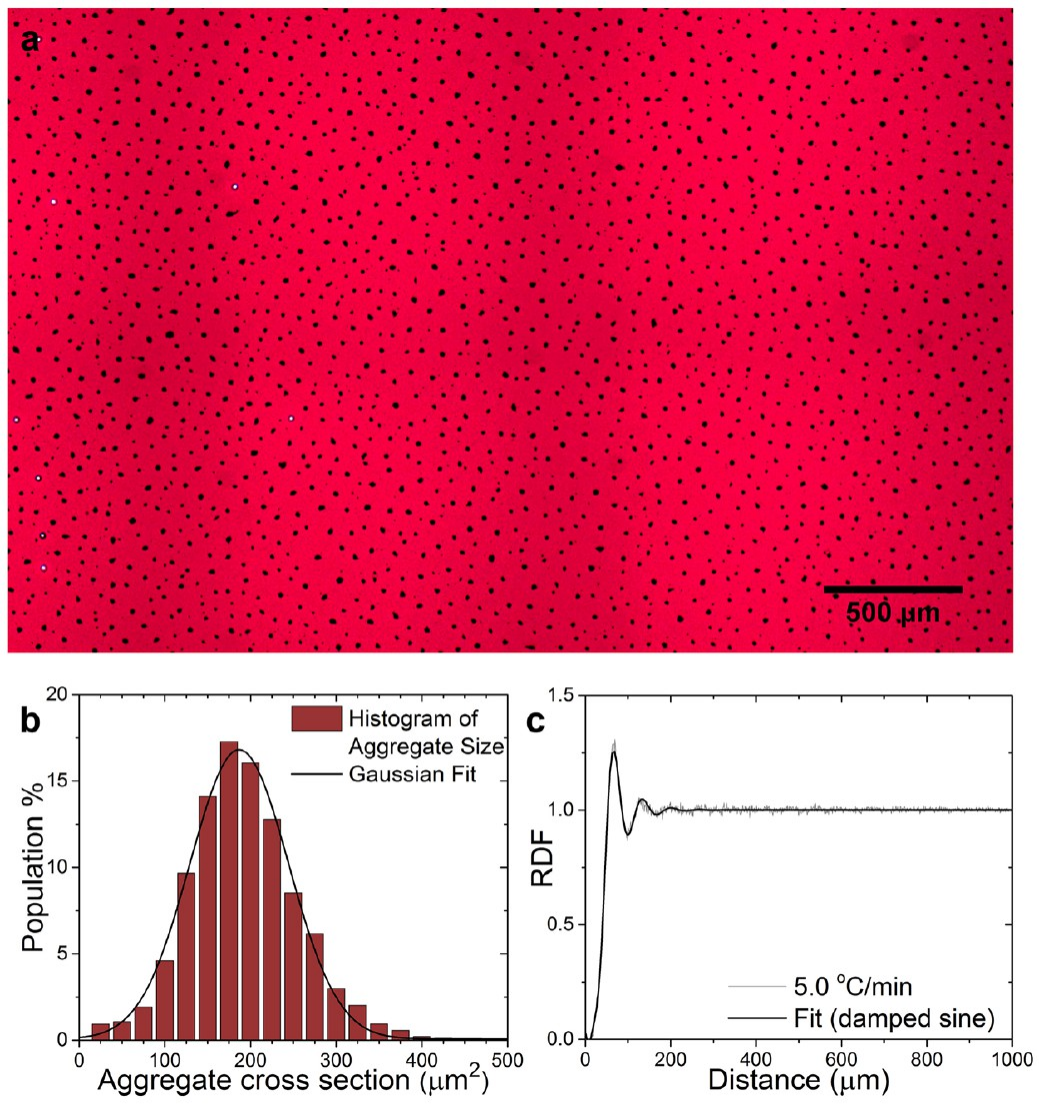
Long-range nanoparticle agglomerate formation. (a) Bright field transmission microscopy image of nematic liquid crystal-nanoparticle composite that was cooled from the isotropic phase to 28 °C at a rate of 5 °C min^–1^. (b) Size distribution of the agglomerates and (c) radial distribution function of the agglomerate positions (center point) fitted with a damped sine function.

In Figure 2a, a long-range bright transmission microscopy image of a nematic composite after cooling at a rate of 5 °C min^–1^ to 28 °C is shown. In order to determine the characteristic spacing between the agglomerates and evaluate ordering, the radial distribution function (RDF) was calculated. Rastering acquisition with a 5× objective allowed analysis of the entire active area of the LC cell (10 × 10 mm^2^), which contained between 1500 and 60 000 agglomerates depending on the cooling rate.^54^ We refer to the Supporting Information for a comprehensive comparison of the RDF with the nearest neighbor distance (NND) approach, in particular on their robustness toward small positional deviations (Supporting Figure S16). As shown in Figure 2c, the nanoscale building blocks formed a pattern of μm-scale agglomerates with characteristic interagglomerate distances of 68 ± 2 μm, evidenced by a periodic fluctuation of the RDF that can be fitted with a modified damped sine function.

**Figure 3:**
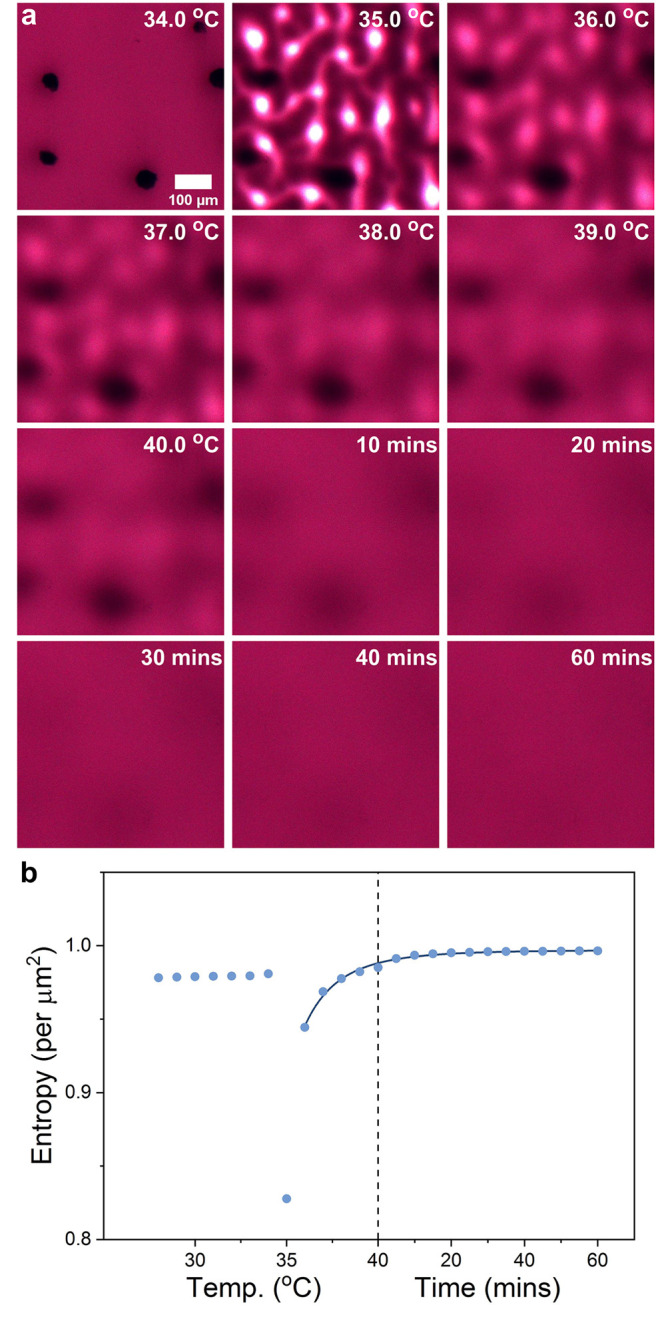
Reversibility of agglomerate array formation. (a) A series of kinetic bright field microscopy images of the composite heated at 1 °/min through the phase transition to 40 ° and held for 60 mins. (b) The information entropy was used to evaluate the homogeneity of the composite as it is heated and maintained in the isotropic phase.

We want to emphasize both the stability of the agglomerates upon formation and the reversibility of the process. Once formed, the NP agglomerates were found to be stable for months only to be redissolved by heating to temperatures above the isotropic phase transition. We did not observe further coarsening of agglomerates or diffusion of NPs in the composite matrix on the time scale of the observation. An analysis of the reversibility of agglomerate formation is shown in Figure 3. To quantify the reversibility, i.e., whether the assembly would fully dissolve upon heating to the isotropic state, each image of the composite, comprising an area of approximately 3000 μm^2^, was converted to a grayscale matrix. The information entropy can serve to evaluate the uniformity of a system, herein for the uniformity of pixel values.^55^ This was conducted following a procedure introduced by our group recently, with results shown in Figure 3b.^56^ For a perfectly homogeneous system, each pixel value should be equivalent, resulting in a maximum entropy per μm^2^ of 1. As the variation in absorbance for each pixel increases (equivalent to a variation in gray scale values), the entropy is reduced. Due to the presence of the glass spacer beads and other minor aberrations in the LC cell and optical path, the upper limit may not be obtained but the reversibility over time can be tracked. Prior to the nematic-to-isotropic phase transition, the entropy per μm^2^ remained relatively constant at an average of 0.979 μm^–2^. At the transition onset, this dropped drastically to 0.828 μm^–2^. With diffusion of the AuNPs into the isotropic phase, the entropy per μm^2^ increased toward 1 following a logistic trend, and, thus, demonstrating homogeneity.

It is interesting to notice that the nature of the steady state may be rather different from the arrested aggregations observed in early work with μm-sized particles.^42,57^ The high solubility of the particles and their small size prevent them from jamming into soft solid structures. Instead, we discerned a dynamic, reversible separation between NP-enriched and NP-depleted regions, characterized by high and low LC order parameters, respectively, more similar to biomolecular condensates. An open question remains, whether this is a real equilibrium state or a long-lived kinetically trapped state.

**Figure 4:**
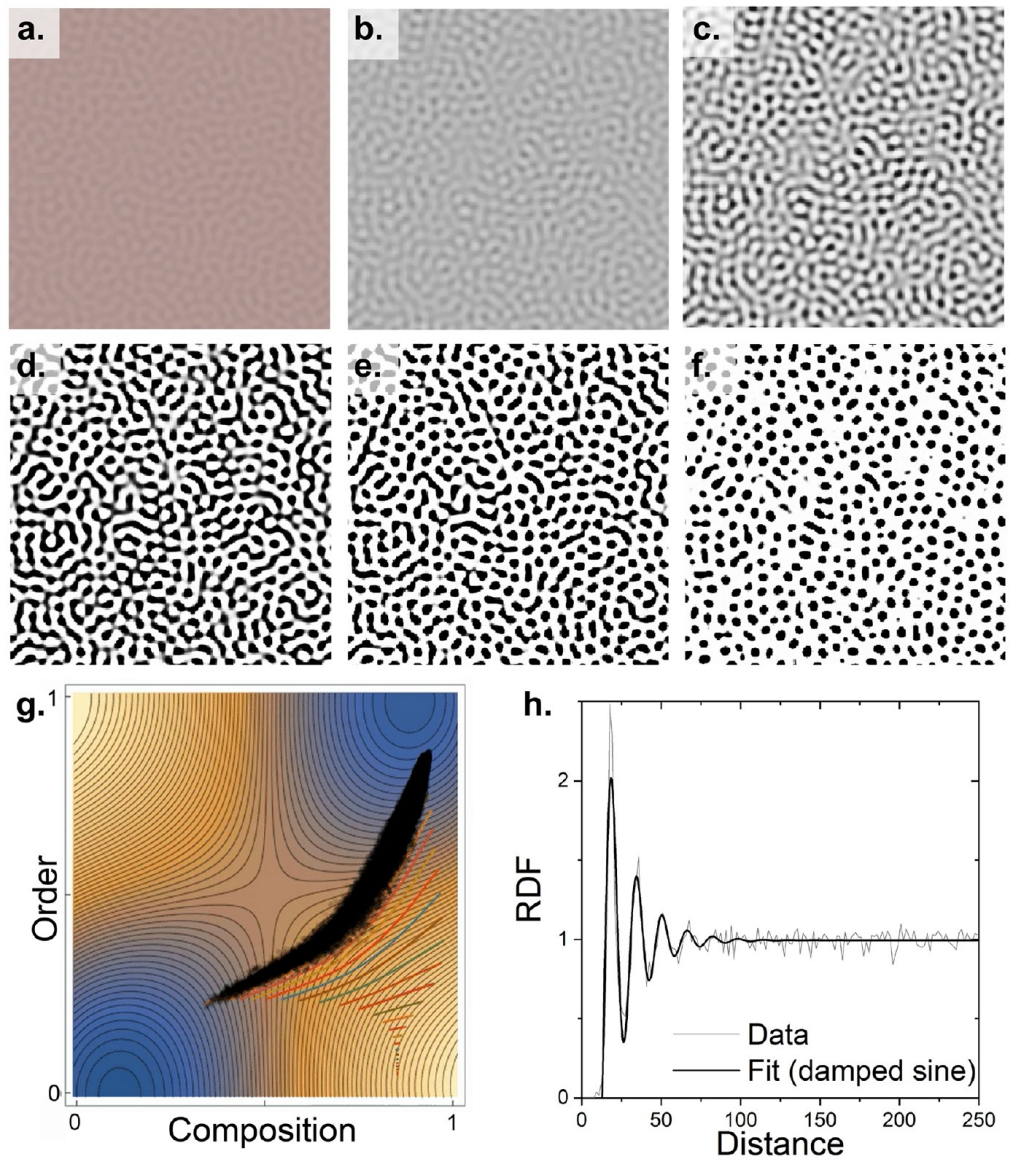
Simulation of NP segregation and agglomerate ordering. (a–f) Sequential snapshots of the evolving microstructure. The grayscale indicates  with black being pure NP. Pink indicates  and is scaled so that small η is more visible. (g) Morphological evolution superimposed on the empirical free-energy landscape. The black dots represent the *c*–η of the microstructure’s “pixels” at a specific time: the extreme points are a superposition of pixels deriving from the homogeneous phases; the (more visible) intermediate points derive from the interface. The colored curves are a data-reduction of pixels and illustrate the character of microstructure evolution. (h) Radial distribution functions obtained by the simulation and an example experiment.

A coarse-grained simulation the NP-mesogen system’s morphological evolution illustrates how an initially homogeneous system can undergo a transient spinodal decomposition followed by a breakup into isolated NP-enriched regions as observed in Figure 1. We performed a phase-field simulation, which couples Cahn–Hilliard equation for the mesogen composition  and an Allen-equation equation for an alignment order parameter  (described below) via an empirical free energy illustrated in Figure 4g. The simulation method’s order-composition coupling derives from Boettinger–Warren–Beckman–Karma,^58^ and further details of the method and the empirical free-energy function appear in the Supporting Information. We define the alignment order-parameter  as the modulus of the dot-product  where  is the local director field of the nematic liquid crystals (or, the largest eigenvector of the Q-tensor order parameter, but this is not explicitly considered here) and *ŝ* is the vector describing the specific orientation induced by the substrate. Where η = 1 implies all domains of mesogens are aligned by the substrate; η = 0 relates to the nonexistence of a substrate effect. For *c* = 1, the system is pure mesogen; *c* = 0 implies it is pure NP. Further details on the phase field simulations approach can be found in the Supporting Information. As shown in Figure 4, the morphological evolution has two stages. Initially, the NPs spontaneously segregate into spinodal laminae with a characteristic wavelength, dominated by the reduction in bulk free-energy. The second stage is dominated by reduction in interfacial energy: the laminae break up and coarsen, resulting in isolated NP-enriched/η-small regions enveloped into a NP-depleted/long-range-ordered phase with a radial-distribution similar to the experimentally observed one. Decomposition initates in the free energy’s spinodal region and eventually the extreme compositions pass into the metastable nucleation and growth region.

In general, it remains extremely difficult to spatially control microstructures formed by liquid–liquid phase separation processes.^59^ The outlined characteristics of structure formation by phase transition are significantly different to conventional spinodal decomposition-based processes that exhibit a conserved order parameter, such as the demixing of polymer blends,^60,61^ sol–gel-polymer^62^ and fullerene-polymer composites,^63^ bicontinuous interfacially jammed emulsion gels,^64,65^ polyelectrolyte multilayers or the electrochemical or liquid metal dealloying.^66,67^ Beyond examples found in nature,^68,69^ some of the most successful synthetic approaches to arrest and control phase separation and therefore the length scale of structure formation rely on mechanical forces to control the kinetics of demixing, e.g., provided by gelation or cross-linking of polymer networks.^70,71^ The approach presented here starts with a homogeneous mixture that is fully isotropic. Cooling induces demixing with NP-enriched nematic microdomains with random alignment and NP-depleted aligned nematic regions. The phase coexistence is only transient and thus, at the end of the process, the composite displays the aligned nematic phase with a characteristic spacing and size of agglomerates depending on the cooling rate.

**Figure 5:**
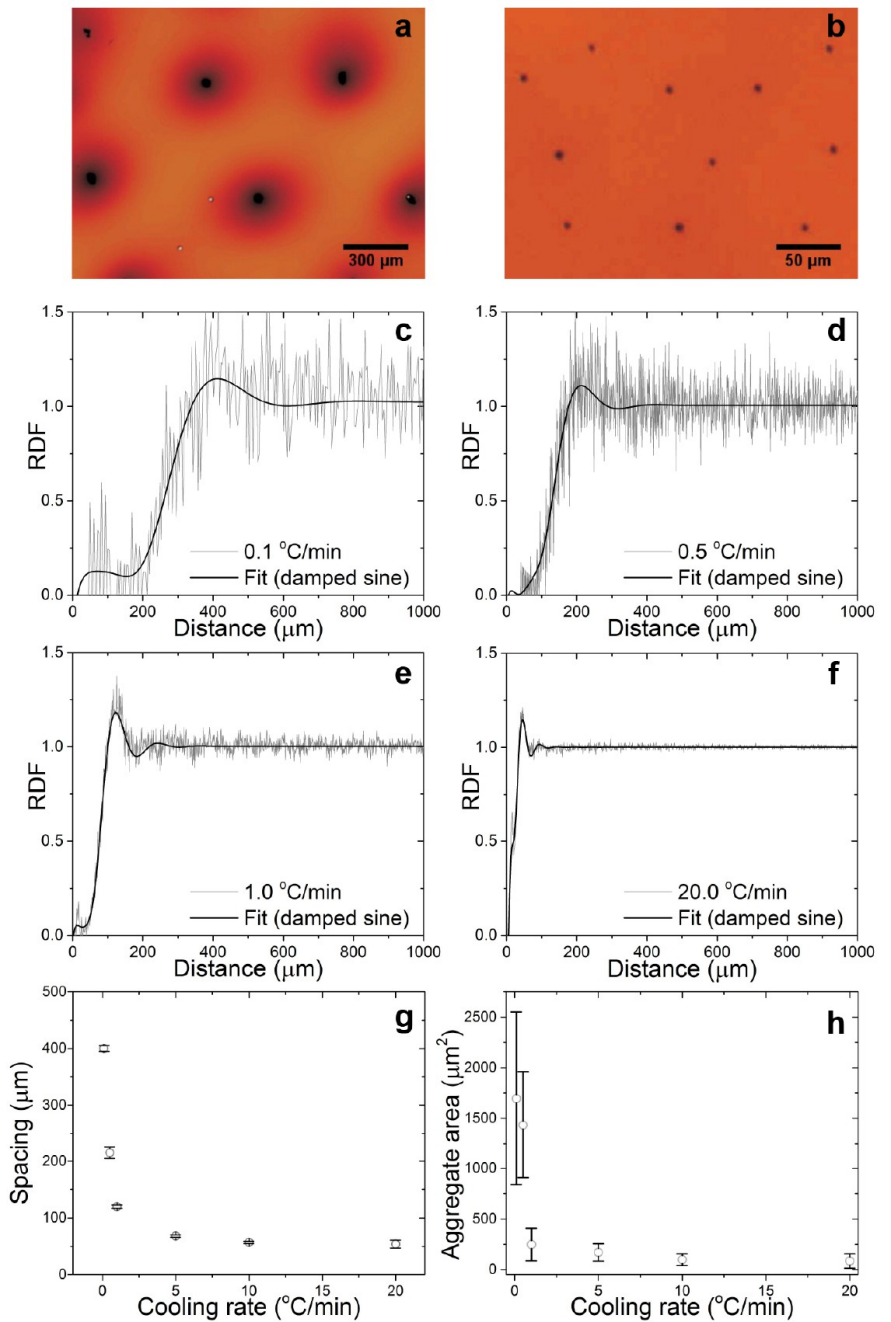
Effect of cooling rate on order parameters. (a,b) Zoom-in of agglomerates resulting from a cooling rate of 0.1 and 20 °C min^–1^, respectively. (c–f) Radial distribution function and corresponding damped sine fit for liquid crystal-nanoparticle composites exposed to different cooling rates. (f) Overview of characteristic spacing as a function of cooling rate. (g) Overview of mean agglomerate size as a function of cooling rate.

The effect of the cooling protocol on the agglomerate ordering is shown in Figure 5. Six different cooling rates, ranging from 0.1 to 20 °C min^–1^ were studied. Samples were cooled to 28 °C and left to equilibrate for 2 h. Between cycles, the LC-NP composite was heated to 50 °C for 2 h to erase the sample history, and the process was repeated multiple times to evidence full reversibility. The corresponding RDFs demonstrate a nonlinear decrease in interagglomerate spacing with increasing cooling rate. Below 1 °C min^–1^, a small increase in the cooling rate produced a significant reduction in the average interagglomerate distance. In contrast, higher cooling rates exhibited a much-diminished dependence. Likewise, the mean agglomerate size was found to correlate with the cooling rate as shown in Figure 5h and in Supporting Figure S17. Note that the increased noise for slower cooling rates is related to the significantly reduced population size considered in the RDF, because fewer agglomerates were formed and these were spaced further apart. We note that the herein described assembly process separates the agglomerates evenly over distances that are significantly larger than the mean agglomerate sizes. We determined for spacing *l* and diameter *d* the dimensionless ratio to be in the range of 8 < *l*/*d* < 18. The domains did not agglomerate over time, and the formation of dimers was only a very rare event.

Based on arguments of mass conservation and from tracking the loss in absorbance for the aligned nematic phase as well as the cross-sectional area of the agglomerates, we calculated an densification factor of NPs within the agglomerates. For a 5 wt % composite cooled at 0.1 °C min^–1^, 91.1% of the AuNPs transfer into the aligned nematic phase, leaving 8.9% in the residual agglomerates. Following the relative fraction of agglomerates by 2D image analysis, one obtains a densification factor of 11.2, which was found to be lower for higher cooling rates. However, in this approximation, the agglomerates were assumed to extend across the entire 20 μm thickness of the LC cell, which may from consideration of their cross-sectional area only be likely for cooling rates below 1 °C min^–1^. Hence, in a sample with an overall 5 wt % concentration, even within the NP agglomerates the LC mesogens retained a volumetric majority, which may explain the reversibility of the process.

The dynamics of the assembly process for different cooling rates was further studied by time-lapse microscopy. Though the principles of agglomerate formation were found to be similar to the observations reported in Figure 1, the process differed not only in the characteristic length scale but also in the characteristic time scale. While the formation of agglomerates was completed in 1.5 min for a cooling rate of 20 °C min^–1^, the whole process required around 100 min at 0.1 °C min^–1^. A comparison of the spinodal-type patterns before breakup of the percolation for the receding NP-enriched phase is shown in Supporting Figure S18 for different cooling rates. The observed characteristic length scale mirrors the characteristic agglomerate spacing after completion of the isotropic-to-nematic phase transition, demonstrating that the NP agglomerates are indeed a remnant of the initial phase separation and can therefore be controlled through parameters that govern this process.^72^

The concentration of NPs in the isotropic phase before cooling played an important role in the overall agglomerate formation. As shown in Supporting Figure S19, reducing the NP concentration to 2.5 wt % led to a decrease in the mean agglomerate size and a less pronounced peak in the RDF. At concentrations below 1.5 wt %, no μm-sized agglomerates were observed in the final aligned nematic composite, most likely due to the fact that the transient local increase in NP concentration in the receding random nematic phase was eventually consumed by NP diffusion into the aligned nematic phase. Note that doping of the LC with Au NPs also led to a small decrease in the observed transition temperature from 34.8 °C for 0 wt %  to 34.3 °C for 5 wt %, which is in line with previous studies that reported an NP-induced dilution effect.^38,40^ Further details on the effect of NP concentration and applied cooling rate on the observed phase transition temperature can be found in Supporting Figures S20 and S21.

**Figure 6:**
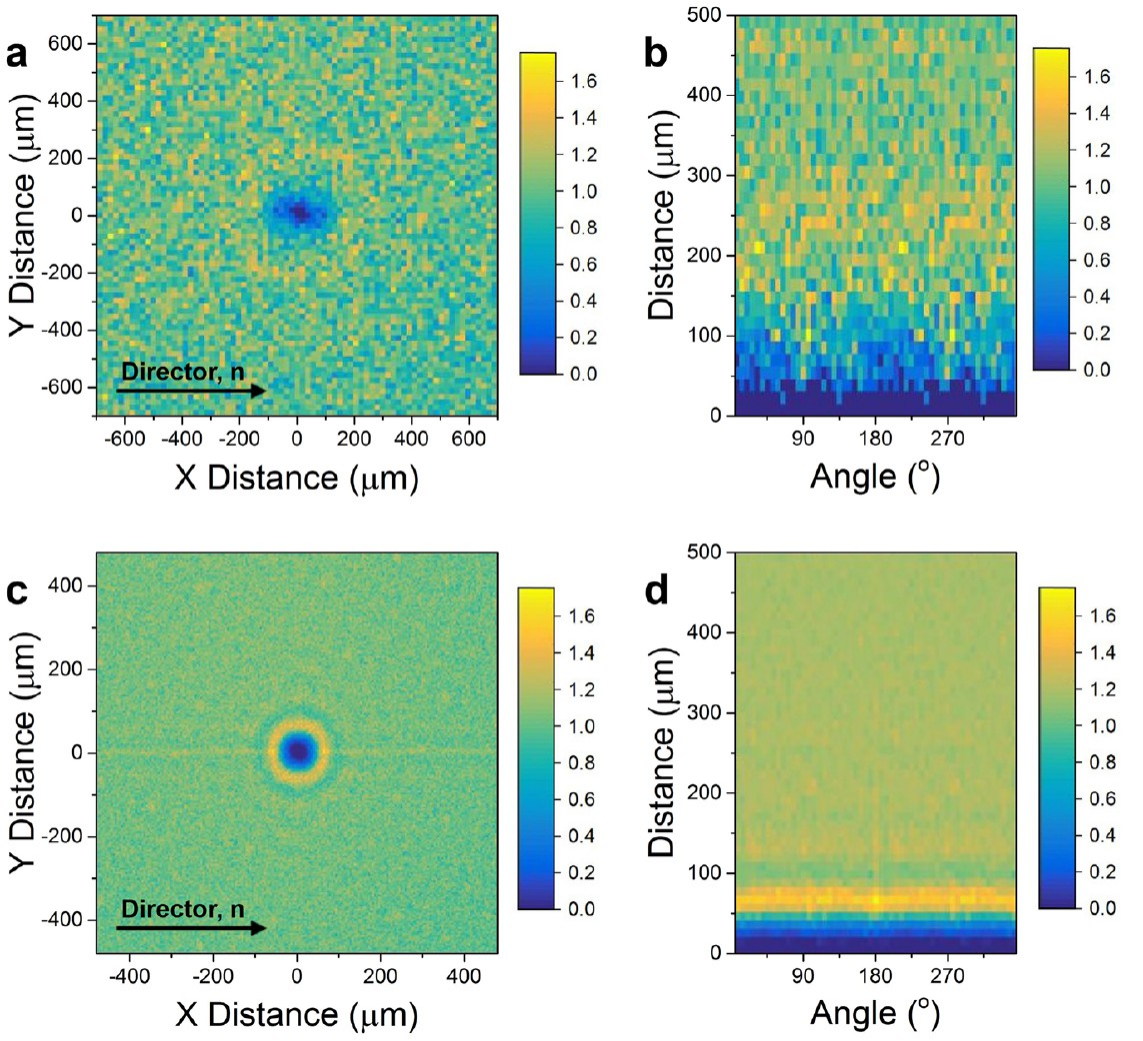
2D orientation of nanoparticle agglomerates in the liquid crystal - nanoparticle composite. 2D histogram of the agglomerate spatial distribution for a cooling rate of 0.5 °C min^–1^ in *x*–*y* coordinates (a) and polar coordinates (b), respectively. (c,d) 2D histogram of the agglomerate spatial distribution for a cooling rate of 5 °C min^–1^.

The most important consequence of this process is the hierarchical organization of NPs into larger assemblies within a nematic composite. The RDF demonstrates positional order of agglomerates over the full radial profile and does, therefore, not account for a possible anisotropy due the nematic phase and alignment along a common director. In Figure 6, 2D histograms of the spatial distribution are shown to represent the agglomerate spacing in *x*–*y* and polar coordinates for 0.5 and 5 °C min^–1^, respectively. Details of the applied methodology,^73^ the experimental results for other cooling rates, as well as a simulation of the effect of disorder on the resulting 2D histograms can be found in Supporting Figures S22 and S23.

At 0.5 °C min^–1^ (Figure 6a), i.e., for larger agglomerates that are further spaced from each other, the agglomerate depletion zone (the region in which the likelihood of finding another agglomerate is below the mean) exhibited an ellipsoidal shape and, thus, a clear anisotropy. In order to measure the eccentricity of this behavior, an angular window was chosen that allowed sufficient statistical evidence for the determination of the characteristic spacing by a segmented radial profile, here 10°. The segmented radial profile along the director field was determined and compared with the segmented radial profile of the respective angular window perpendicular to the director field. Based on the characteristic spacing obtained in both directions, an eccentricity of 0.71 ± 0.02 was found. The spatial modulation as a function of the relative orientation to the mesogen director field is directly evident in the polar coordinate plot shown in Figure 6b. At higher cooling rates and thus smaller agglomerates with smaller spacing (5 to 20 °C min^–1^), an anisotropy in the depletion zone was not observed. Instead, an increased probability of finding agglomerates along the director field became evident, discernable in Figure 6c as a yellow line along the *Y* = 0 direction and in Figure 6d as an increased probability around the 180 ° window. A segmented radial profile over an angular window of 1 ° in the direction of director field was compared to that around 45 ° and 90 ° with respect to the direction of the director field. Based on the results obtained herein, for a cooling rate of 5 °C min^–1^, the probability of finding another agglomerate along the director field was 9% higher than in any other direction. A similar trend was observed for cooling rates of 10 and 20 °C min^–1^.

## Conclusions

In summary, we report on the reversible collective assembly of gold NPs in a thermotropic LC driven by the phase transition from the isotropic to the nematic phase under anchoring-driven planar alignment. Based on our observations, the phase dependent solubility, cooling temperature and homogeneous alignment by the substrate play a decisive role in the formation of hierarchically structured LC-NP composites with NP agglomerates of controllable size and characteristic spacing. While reduced solubility in the unidirectionally aligned nematic phase results in residual NPs to be expelled in agglomerates, their spacing depends on the cooling rate that defines a characteristic length scale in the compartmentalization through the imposed transient decomposition process during phase transition. Once formed, the agglomerates were found to be stable for months and the process remained reversible by heating above the nematic-to-isotropic phase transition temperature. Phase field simulations, where the nematic ordering is determined by an Allen–Cahn model and the NP concentration is governed by a Cahn–Hilliard model, exhibit a similar evolution of the microstructural characteristics as the experimental findings. As a model platform, this materials system provides insights into the control over the size and spatial arrangement of hierarchical microstructures from nanoscale building blocks formed by liquid–liquid phase separation.^59^ The observation of controlled and phase transition-driven reversible assembly of gold NPs takes inspiration from other forms of stimuli-directed self-assembly,^74^ where the stimuli may be a redox reaction, solvent addition, pH change, or light exposure.^75,76^ The presented findings of such a controlled assembly process may yield interesting opportunities toward programmable, switchable and reconfigurable dynamic photonic materials for sensing, spatial light modulation and display applications.^77^ When applied to quantum dot materials, one may expect suitable properties toward reconfigurable lasers.^78^ Furthermore, in case the transition can be triggered by the system itself, e.g., by light absorption, this principle of structure formation may enable further exploration of active soft matter.^79,80^

## Experimental Section

### Ligand Synthesis

The ligand synthesis was carried out following an adapted literature procedure from Milette and co-workers.^37^

#### General Procedures

Unless otherwise noted, all reactions were carried out in dried Schlenk glassware in an inert argon atmosphere. Chromatography solvents were purchased as reagent grade and distilled once prior to use. For reactions, dichloromethane (DCM), methanol (MeOH), tetrahydrofuran (THF), and *N*,*N*-dimethylformamide (DMF) were purchased dry over molecular sieves from Acros Organics, and acetone was purchased dry from Sigma-Aldrich. All reagents were commercially obtained and used without further purification. 4′-Hydroxy-4-biphenylcarbonitrile (99%) and hexamethyldisilthiane (97%) were purchased from ABCR, 1,12-dibromododecane (98%) was purchased from TCI, potassium thioacetate (98%) was purchased from Alfa Aesar, and acetyl chloride (ACS reagent grade), anhydrous potassium carbonate (99%), and tetrabutylammonium fluoride (1 M solution in THF) were purchased from Acros Organics. TLC analyzes were performed on TLC plates from Merck (Silica gel 60 F_254_). UV-light (254 nm) or anisaldehyde staining was used for detection. Column chromatography was conducted on Geduran silica gel Si 60 from Merck (40–60 μm).

#### 4′-(12-Bromododecyloxy)-4-biphenylcarbonitrile **1**

4′-Hydroxy-4-biphenylcarbonitrile (4.38 g, 22.4 mmol) and 1,12-dibromododecane (36.82 g, 112.2 mmol) were dissolved in dry acetone (750 mL). Potassium carbonate (6.11 g, 44.9 mmol) was added, and the mixture was heated to reflux for 10 h, after which 4′-hydroxy-4-biphenylcarbonitrile was consumed according to TLC (DCM). After dilution with DCM (300 mL) the mixture was washed twice with 1 M HCl and once with saturated NaCl solution. The organic phase was dried over MgSO_4_, and concentrated in vacuo. Column chromatography (silica gel; DCM/*n*-heptane 1:2) afforded 4′-(12-bromododecyloxy)-4- biphenylcarbonitrile **1** (8.69 g, 19.6 mmol, 88%) as a colorless powder. ^1^H NMR (400.13 MHz, CDCl_3_): δ = 7.70–7.63 (m, 4H, Ph*H*), 7.54–7.51 (m, 2H, Ph*H*), 7.01–6.97 (m, 2H, Ph*H*), 4.01 (t, *J* = 6.5 Hz, 2H, C*H*_2_OPh), 3.41 (t, *J* = 6.9 Hz, 2H, C*H*_2_Br), 1.89–1.77 (m, 4H, 2 C*H*_2_), 1.51–1.29 (m, 16H, 8 C*H*_2_). ^13^C NMR (100.61 MHz, CDCl_3_): δ = 159.9, 145.4, 132.7, 131.4, 128.4, 127.2, 119.3, 115.2, 110.2 (8 Ph*C*, 1 *C*N), 68.3 (*C*H_2_OR), 34.2 (*C*H_2_Br), 33.0, 29.7, 29.7, 29.6, 29.6, 29.5, 29.4, 28.9, 28.3, 26.2 (10 *C*H_2_).

#### *S*-(12-(4′-(4-Biphenylcarbonitrile)oxy)dodecyl)ethanethioate **2**

4′-(12-Bromodo-decyloxy)-4-biphenylcarbonitrile **1** (0.98 g, 2.2 mmol) was added to a dispersion of *S*-potas-sium thioacetate (1.25 g, 11.0 mmol) in DMF (30 mL) and the mixture was heated to 70 °C for 12 h. After dilution with DCM (150 mL) the mixture was washed six times with 1 M HCl and once with saturated NaCl solution. The organic phase was dried over Na_2_SO_4_, and concentrated in vacuo. Column chromatography (silica gel; DCM/*n*-heptane 5:1) afforded *S*-(12-(4′-(4-biphenylcarbonitrile)oxy)dodecyl) ethanethioate **2** (0.91 g, 2.1 mmol, 94%) as an off-white solid. ^1^H NMR (400.13 MHz, CDCl_3_): δ = 7.70–7.63 (m, 2H, Ph*H*), 7.54–7.51 (m, 2H, Ph*H*), 7.00–6.98 (m, 2H, Ph*H*), 4.00 (t, *J* = 6.6 Hz, 2H, C*H*_2_OPh), 2.86 (t, *J* = 7.4 Hz, 2H, C*H*_2_SAc), 2.32 (s, 3H, C*H*_3_), 1.81 (dt, *J* = 14.6, 6.7 Hz, 2H, C*H*_2_CH_2_OPh), 1.60–1.52 (m, 2H, C*H*_2_CH_2_SAc), 1.50–1.43 (m, 2H, 1 C*H*_2_), 1.35–1.28 (m, 14H, 7 C*H*_2_). ^13^C NMR (100.61 MHz, CDCl_3_): δ = 196.2 (CH_3_*C*OS), 160.0, 145.4, 132.7, 131.4, 128.5, 127.2, 119.3, 115.2, 110.2 (8 Ph*C*, 1 *C*N), 68.3 (*C*H_2_OR), 30.8 (*C*H_3_COS), 29.7, 29.7, 29.6, 29.5, 29.4, 29.3, 29.3, 29.0, 26.2 (11 *C*H_2_).

#### 4′-(12-Mercaptododecyloxy)-4-biphenylcarbonitrile **3**

##### From S-(12-(4′-(4-Biphenylcarbonitrile)oxy)dodecyl)ethanethioate **2**

*S*-(12-(4′-(4-Biphenyl-carbonitrile)oxy)dodecyl)ethanethioate **2** (440 mg, 1.0 mmol) was dissolved in dry DCM (7 mL), and dry methanol (10 mL) was added. The mixture was stirred at room temperature, acetyl chloride (0.5 mL, 7.0 mmol) was added dropwise, and stirring was continued for 6 h. After dilution with DCM (30 mL) the mixture was washed once with saturated NH_4_Cl solution and once with saturated NaCl solution. The organic phase was dried over Na_2_SO_4_, and concentrated in vacuo. Column chromatography (silica gel; DCM) afforded 4′-(12-mercaptododecyloxy)-4-biphenylcarbonitrile **3** (183 g, 0.46 mmol, 46%) as an off-white solid.

##### From 4′-(12-Bromododecyloxy)-4-biphenylcarbonitrile **1**

4′-(12-Bromododecyloxy)-4-biphenylcarbonitrile **1** (5.00 g, 11.3 mmol) was dissolved in dry THF (100 mL), and the solution was cooled to 0 °C. Hexamethyldisilathiane (2.85 mL, 13.56 mmol) was added dropwise to the solution, followed by dropwise addition of tetrabutylammonium fluoride (12.4 mL, 1.0 m in THF, 12.43 mmol). The reaction was allowed to warm up to room temperature and stirred for 2 h. After dilution with DCM (200 mL) the mixture was washed twice with saturated NH_4_Cl solution and once with saturated NaCl solution. The organic phase was dried over Na_2_SO_4_, and concentrated in vacuo. Column chromatography (silica gel; DCM/*n*-heptane 1:1) afforded 4′-(12-mercaptododecyloxy)-4-biphenylcarbonitrile **3** (3.30 g, 8.3 mmol, 74%) as colorless solid. ^1^H NMR (400.13 MHz, CDCl_3_): δ = 7.70–7.63 (m, 4H, Ph*H*), 7.54–7.51 (m, 2H, Ph*H*), 7.01–6.97 (m, 2H, Ph*H*), 4.00 (t, *J* = 6.5 Hz, 2H, C*H*_2_OR), 2.52 (q, *J* = 7.4 Hz, 2H, C*H*_2_SH), 1.81 (dt, *J* = 14.6, 6.7 Hz, 2H, C*H*_2_CH_2_OR), 1.64–1.57 (m, 2H, C*H*_2_), 1.51–1.43 (m, 2H, C*H*_2_) 1.37–1.28 (m, 14H, 7 C*H*_2_). ^13^C NMR (100.61 MHz, CDCl_3_): δ = 160.0, 145.4, 132.7, 131.4, 128.5, 127.2, 119.3, 115.2, 110.2 (8 Ph*C*, 1 *C*N), 68.3 (*C*H_2_OR), 34.2, 29.7, 29.6, 29.5, 29.4, 29.2, 28.5, 26.2, 24.8 (11 *C*H_2_).

### Nanoparticle Synthesis

Oleylamine AuNPs were synthesized as follows.^50^ A precursor solution containing 10 ml *n*-octane (Sigma-Aldrich, puriss.), 10 ml oleylamine (Acros, C18 80–90%) and 0.25 mmol HAuCl_4_. 3H_2_O (Sigma-Aldrich, 99.9+% metals basis) was prepared and stirred under inert atmosphere at a temperature of 15 °C, which was controlled with 0.1 K precision. Separately, 0.25 mmol of the reducing agent t-butylamine borane (Strem, 97%+) was dissolved in a solvent mixture of 1 ml *n*-octane and 1 ml oleylamine. Subsequently, the reducing solution was injected quickly into the precursor solution and left stirring at for 1 h. The oleylamine-protected AuNPs were subsequently 2× washed in ethanol (Fluka, HPLC grade) with a minimal amount of DCM (Carlo Erba, ACS grade) and subsequently redispersed in DCM. Ligand exchange was carried out by preparing a thiol solution containing 15 ml DCM with 0.127 mmol of 1-hexanethiol (Alfa Aesar, 97%+) and 0.085 mmol of MDD-CBO, i.e., a 60/40 mol % mixture. Subsequently, a solution with 25 mg oleylamine-protected AuNPs and 5 ml DCM was added and left stirring for 24 h at room temperature. AuNPs were cleaned by dispersing them in a 10/90 vol % mixture of DCM and acetone (Sigma-Aldrich, puriss.) and subsequent precipitation by ultracentrifugation (32,000 rpm, 1 h). This step was repeated 3 times. A mixture of 10/10/80 vol % tetrahydrofuran, acetonitrile (Carlo Erba, HPLC grade), and acetone (Sigma-Aldrich, puriss.) was used for two subsequent cleaning cycles. We want to note that usual protocols such as the repeated precipitation in a poor solvent (here: acetone) and subsequent centrifugation (at 5,000 rpm for 10 min) as well as cleaning by a Soxhlet extractor did not result in an NMR signal that is dominated by surface-bound ligands.

### Sample Fabrication

AuNP-LC composites were fabricated according to a protocol published by Qi and Hegmann.^51^ In short, thiol-protected AuNPs were dissolved in DCM and mixed in the targeted wt % with the LC (5CB; 4-cyano-4′-pentylbiphenyl, Synthon, 99.8%). The mixture was stirred then sonicated for 1 min before the volatile components were evaporated overnight at 60 °C under a stream of nitrogen (Eppendorf ThermoMixer C). Subsequently, the solution was transferred to a vacuum oven (Heraeus Vacutherm), which was set to 50 °C for 3 h to remove any remaining traces of solvent. Once complete, the sample was sealed and stored in the thermomixer at 40 °C until use.

Subsequently, the AuNP-LC composite was infiltrated in a glass sandwich with defined gap thickness and surface functionalization. Cells with a homogeneous or homotropic surface alignment and 4–20  μmthickness were supplied by Instec Inc. Reference cells without surface alignment were built with precleaned microscope slides where an initially 25 μm thick thermoplastic sealing film (DuPont Surlyn, Meltonix 1170–25) served as spacer. Prior to infiltration, the cell was placed on the temperature controlled stage and warmed to 40 °C. Once the cell had been heated, 10 μL of the AuNPLC composite was taken from the vial and slowly deposited on the cell near the opening. Capillary action drew the composite into the cell. The cell was left at 40 °C for at least 15 min to allow a uniform film to form inside.

### Optical Microscopy

Optical microscopy was carried out in transmission on an Olympus BX61. The following objectives were used: 5× (UMPlanFI, NA 0.15), 10× (UMPlanFI, NA 0.30), and 20× (UMPlanFI, NA 0.46). The samples were temperature controlled with a Peltier-driven hot stage (Linkam, PE120). The effective temperature within the LC cell was determined by a calibration run.

### Microspectroscopy

Spectroscopic absorption measurements were carried out on the BX61 with a 20× magnifying objective and the in-built halogen lamp (100 W). The signal was collected through a microspectroscopy port with a 200 μm fibre and an Ocean Optics QE 65000 spectrometer. This resulted in a collection spot size of around 25  μm.

### Nuclear Magnetic Resonance (NMR) Spectroscopy

NMR experiments were carried out at 297.2 K on a Bruker Avance III 400 spectrometer at frequencies of 400.13 MHz for ^1^H nuclei and 100.62 MHz for ^13^C nuclei or on a Bruker Avance 400 spectrometer with a BBIz 5 mm probe at a frequency of 400.13 MHz for ^1^H nuclei. Spectra were calibrated to the residual solvent peak of CDCl_3_ (7.26 ppm ^1^H NMR; 77.16 ppm ^13^C NMR).^81^

### Transmission Electron Microscopy (TEM)

TEM was carried out on a Philips/FEI CM12 with a LaB_6_ source that was operated at 120 kV accelerating voltage. Size analysis was carried out using imageJ.

### Free Energy Modeling with Coupled Allen–Cahn and Cahn–Hilliard Equations

The relevant equations are given by^82^
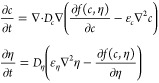
1where the composition (*c*) and order (η) are understood to be time-dependent fields: , . *D*_*c*_ and *D*_η_ set the time scales for the diffusion of NP and the rate of ordering of the mesogens and are assumed to be constant in the simulations. Interfaces separating two regions of differing compositions are associated with large |∇*c*| and the width of that interface scales with  (ϵ_*c*_ is the square-gradient coefficient), where *f*_max_ is maximum value of the free energy *f*(*c*, η) in the interfacial region. The interfacial tension scales as . Scaling for the order–disorder interface is the same but with subscripts changed. In the simulation, an initial uniform composition and order were chosen as initial conditions to which a small amount of noise was added. The two eqs 1 were then updated in a leapfrog process: one field is updated with its own value at −Δ*t* and the other’s value at −Δ*t*/2. A semi-implicit spectral method was employed.^83^

## Supporting Information Available

Details of coarse-grained molecular dynamics simulations; electron microscopy of gold nanoparticles; schematic of ligand synthesis; ^1^H and ^13^C NMR spectra of ligand synthesis; ^1^H NMR spectra of supernatant from nanoparticle washing; 1H spectra of NPs before and after etching; UV–vis spectroscopy of NPs in various solvents; LC isotropic–nematic phase transition under cross-polarization; LC nematic–isotropic phase transition under cross-polarization; Molar attenuation coefficient (ϵ) of the AuNPs in the isotropic and nematic phase; nearest neighbor distance vs radial distribution function of model arrays; analysis of effect of cooling rate on the agglomerate size; UV–vis spectroscopy of nematic LC-NP composites from different cooling protocols; effect of cooling rate on characteristic length scale of initial phase decomposition; effect of AuNP concentration on characteristic agglomerate area and distancing; DSC analysis of 5CB and AuNP-LC composite; phase transition temperature of the AuNP-LC composite; 2D alignment of AuNP agglomerates in the LC effect of cooling rate (experimental); 2D alignment of AuNP agglomerates in the LC effect of disorder (simulation); effect of homogeneous surface alignment under cross-polarization; LC nematic–isotropic phase transition under cross-polarization without surface alignment (PDF)
Video of the isotropic–nematic transition under cross-polarization (MP4)

## Notes

Preprint available at: N. Mac Fhionnlaoich, S. Schrettl, N.B. Tito, Y. Yang, M. Nair, L.A. Serrano, K. Harkness, P.J. Silva, H. Frauenrath, F. Serra, W.C. Carter, F. Stellacci, S. Guldin. Reversible Microscale Assembly of Nanoparticles Driven by the Phase Transition of a Thermotropic Liquid Crystal, *ChemRxiv*, 2022. 10.26434/chemrxiv-2021-c93dp-v3 (accessed May 10, 2023).
