# Supplementary material for: Reversible Microscale Assembly of Nanoparticles Driven by the Phase Transition of a Thermotropic Liquid Crystal
Source: ACS Nano. 2023 May 24;17(11):9906–18. doi: 10.1021/acsnano.2c09203 (PMC10278172; doi:10.1021/acsnano.2c09203)
Supplement: Supplementary file 1 — nn2c09203_si_001.pdf [file nn2c09203_si_001.pdf]

—— SUPPORTING INFORMATION ——————

Reversible microscale assembly of nanoparticles driven by the phase transition of a thermotropic liquid crystal

Niamh Mac Fhionnlaoich[1], Stephen Schrettl[2,†], Nicholas B. Tito[3], Ye Yang[1], Malavika Nair[2,‡], Luis A. Serrano[1], Kellen Harkness[2], Paulo Jacob Silva[2], Holger Frauenrath[2], Francesca Serra[4], W. Craig Carter[5], Francesco Stellacci[2], Stefan Guldin[1⋆]

[1]*Department of Chemical Engineering, University College London, London, WC1E 7JE, United Kingdom.* [2]*Institute of Materials, École Polytechnique Fédérale de Lausanne, 1015 Lausanne, Switzerland.* [3]*Eindhoven University of Technology, Department of Applied Physics and Science Education, 5612 AP Eindhoven, The Netherlands.* [4]*Department of Physics, Chemistry and Pharmacy, University of Southern Denmark, 5230 Odense, Denmark.* [5]*Department of Materials Science and Engineering, Massachusetts Institute of Technology, Cambridge, MA 02139, USA.* [†]*Now at: Technical University of Munich, School of Life Sciences, 85354 Freising, Germany.* [‡]*Now at: Institute of Biomedical Engineering, University of Oxford, Headington, OX3 7DQ, United Kingdom* [⋆]*e-mail: S.Guldin@ucl.ac.uk*

METHODS

Coarse-grained molecular dynamics simulations

Coarse-grained molecular dynamics simulations were carried out to examine the free energy of solvating a ligand-coated nanoparticle (collectively called a "complex" here) into isotropic and nematic liquid crystalline phases, respectively. The simulation system is a cubic box with volume V that can fluctuate, under constant pressure P and temperature T via the Martyna-Tobias-Klein barostat-thermostat.[1] The system boundaries are periodic.

Simulation parameters and quantities are all given in terms of fundamental reduced units of distance $\mathcal{D}$, energy $\mathcal{E}$, mass $\mathcal{M}$, and time $\tau = \sqrt{\mathcal{M}\mathcal{D}^2/\mathcal{E}}$. Temperature is given in terms of $k_B T_{\text{real}}/\mathcal{E}$, where k_B is Boltzmann's constant. Calculations were carried out using the HOOMD-Blue molecular dynamics package (v2.1.1).[2,3]

To compute the free energy of solvation, we construct a bulk liquid crystal containing just one complex. The liquid crystal is represented by stiff rod-like molecules, each comprising 4 Lennard-Jones spheres connected together into a string by stiff harmonic bonds and angle potentials. The complex is represented by a large repulsive Lennard-Jones sphere coated with stiff mobile ligands. The latter are similar in structure to the liquid crystal molecules. To calculate the free energy of solvation in an isotropic or nematic phase of the liquid crystal, we perform thermodynamic integration.

Simulation approach, potentials, and parameters The molecular potentials governing the intermolecular, bonding, and angle interactions in the system are as follows. Pair-wise intermolecular interactions are given by the Lennard-Jones potential:

$$U_{LJ}(r) = 4\epsilon\left[\left(\frac{\sigma}{r}\right)^{12} - \alpha\left(\frac{\sigma}{r}\right)^{6}\right] + C_{\text{shift}}, \tag{1}$$

where ϵ and σ are the tunable potential strengths and widths, respectively, and α can be used to independently tune the attractive piece of the potential. The potential is truncated at r_{cut}; for larger r, the potential is zero. The constant C_{shift} is defined so that $U_{LJ}(r_{\text{cut}}) = 0$. In all cases, $r_{\text{cut}} = 2.5\sigma$.

Table 1 lists the Lennard-Jones parameters for the system. All systems have $\epsilon = 1\mathcal{E}$, and all potentials are repulsive (i.e. $\alpha = 0$). The exception is the interaction between liquid crystal molecules and ligands, which have $\alpha = 0.1$ to permit slight favourable (van der Waals) attractions between those species.

	Liquid Crystal Particle	Nanoparticle	Ligand Particle
Liquid Crystal Particle	$\epsilon = 1, \sigma = 1, \alpha = 0$	$\epsilon = 1, \sigma = R_{\text{NP}} + 0.5, \alpha = 0$	$\epsilon = 1, \sigma = 1, \alpha = 0.1$
Nanoparticle			$\epsilon = 1, \sigma = R_{\text{NP}} + 0.5, \alpha = 0$
Ligand Particle			$\epsilon = 1, \sigma = 1, \alpha = 0$

Supplementary Table S1 | Lennard-Jones parameters for the intermolecular interactions in the system. Units of measure are $\mathcal{E}$ for ϵ, $\mathcal{D}$ for σ, and α is dimensionless. The quantity R_{NP} is the effective radius of the nanoparticle, specified in text.

The 4 Lennard-Jones spheres comprising a liquid crystal molecule or ligand are attached via harmonic bonds following the potential

$$U_{\text{bond}}(r) = \frac{1}{2}k(r - r_0)^2, \tag{2}$$

where k and r_0 are the bond spring constant and equilibrium length, respectively. For the liquid crystal molecules we use $k = 5000\mathcal{E}/\mathcal{D}^2$ and $r_0 = 0.5\mathcal{D}$, whereas for the ligands we choose a weaker potential with $k = 400\mathcal{E}/\mathcal{D}^2$ and $r_0 = 0.8\mathcal{D}$. This is to emulate the fact that the ligands are grafted to the nanoparticle surface by somewhat flexible linker species.

The angle potential between triplets of adjacent particles in a liquid crystal molecule or ligand is

$$U_{\text{angle}}(\theta) = \frac{1}{2}\kappa(\theta - \pi)^2, \tag{3}$$

where κ is the angle stiffness constant. For both the liquid crystal molecules and ligands, we use a stiff potential with $\kappa = 200\mathcal{E}/\text{rad}^2$, so that the 4 Lennard-Jones spheres comprising the molecule remain linear and rod-like.

The nanoparticle is represented by a single large repulsive Lennard-Jones sphere, having an effective radius of $R_{\text{NP}} = 5\mathcal{D}$. Ligands are tethered by one end to the center of the nanoparticle by stiff harmonic bonds with $k = 5000\mathcal{E}/\mathcal{D}^2$ and $r_0 = (0.5 + R_{\text{NP}})\mathcal{D}$. The ligands are mobile on the surface of the nanoparticle.

The alignment of liquid crystal molecules in the system is modulated by an external electric field along the x axis of the system. Each liquid crystal molecule is given equal but opposite charges of unity at their two terminal particles. The interaction of the molecule with the external electric field, based on its x coordinate in the system and charge q, is

$$U_E(x) = -qEx, \tag{4}$$

where E is the magnitude of the imposed field in units of $\sqrt{\mathcal{E}/(4\pi\epsilon_0\mathcal{D}^3)}$, and ϵ_0 is the permittivity of vacuum. The magnitude can be varied to obtain different degrees of liquid crystal alignment. (Note that the system does not incorporate any Coulombic interactions between charged particles; the charge serves only to cause interaction with the imposed electric field.)

Systems are defined containing 10000 liquid crystal molecules, and one complex. The complex consists of one nanoparticle, and 220 bound ligands. These particles are randomly positioned in a box of initial volume $(45\mathcal{D})^3$. Systems are first equilibrated in the NVE ensemble for 1×10^4 time steps. Following that, they are equilibrated in the NPT ensemble for 1×10^6 steps. Production statistics are then obtained in the NPT ensemble for 1×10^7 additional steps. Snapshots and statistics are collected at intervals of 1×10^4 time steps. All systems are integrated using a time step size of $dt = 0.001\tau$. In the NPT simulations, $T = 1$ and $P = 12.7\mathcal{E}/\mathcal{D}^3$. At this pressure and temperature, the liquid crystal remains isotropic in the simulation. A nematic (or smectic) phase can be induced by choosing a non-zero value of external electric field strength E. In this study, we obtain a nematic phase by choosing $E = 2$ units, while $E = 0$ units is used for the isotropic phase.

The snapshots shown in Supplementary Fig. 1a and b represent cross-sectional cuts of the three-dimensional simulation, revealing the NP/ligand complex (yellow/red) and the surrounding liquid crystal molecules (blue) in the isotropic and nematic phase, respectively. For simplicity, the ligand shell consisting of short spaceholder and longer mesogen-like ligands was represented by a partial coverage of a soft NP core with an occupied surface area of approximately 55%. A nematic orientational order parameter of $\langle P_2 \rangle = 0.56$ was observed in the nematic phase. In both cases, the simulations indicate that the mesogens of the bulk LC phase interdigitate with the ligand corona.

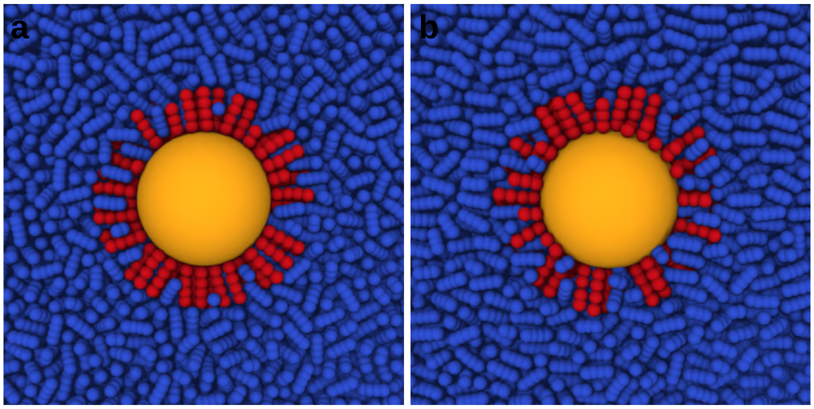

Supplementary Figure S1 | Molecular dynamics simulations of nanoparticle solvation. Cross-sectional snapshots of the nanoparticle complex in the isotropic (**a**) and nematic (**b**) phase. For simplicity, the mixed ligand shell was represented by a partial coverage of a soft NP core with mesogen-like ligands.

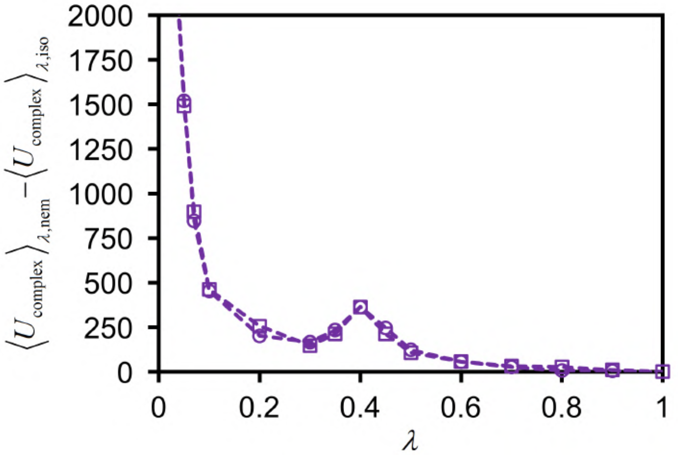

Supplementary Figure S2 | Plot of the difference of the integrand in Eq. 6 between the isotropic and nematic phase, as a function of the thermodynamic integration parameter λ. Open points are simulation averages (circles and squares for first and second simulation replicates, respectively), and dashed lines are guides for the eye.

Thermodynamic integration To compute the free energy of solvating a complex into the isotropic or nematic liquid crystal phase, we employ thermodynamic integration.[4] A continuous thermodynamic pathway is defined from a reference state in which the complex is absent from the liquid crystal, to one in which it is within the liquid crystal. To accomplish this, a "linking potential" U_{link} is defined for the Lennard-Jones interaction between the complex and the liquid crystal. We choose this linking potential to be

$$U_{\text{link}}(\lambda) = \lambda U_{\text{complex}}. \tag{5}$$

where λ is the scaling variable, and U_{complex} is defined as the sum of the *full* Lennard-Jones interactions of all liquid crystal molecules with: the nanoparticle core; and the ligand particles. The free energy of solvation is then computed by integrating

$$F_{\text{solv}} = \int_0^1 \left\langle \frac{dU_{\text{link}}(\lambda)}{d\lambda} \right\rangle_\lambda d\lambda = \int_0^1 \langle U_{\text{complex}} \rangle_\lambda d\lambda. \tag{6}$$

This integral is computed numerically by performing a series of simulations at various values of λ. For a given λ, the complex interacts with the liquid crystal via the *scaled* potential $\lambda U_{\text{complex}}$; however, we record the average *full* potential energy U_{complex} during the simulation. This leads to an ensemble-averaged value of the full U_{complex}, but within the ensemble with the λ-scaled potential $\lambda U_{\text{complex}}$, as required by Eq. 6.

Equation 6 can be applied to determine the solvation free energy in both the isotropic and nematic phase. Of interest is the *difference* between these two quantities, i.e.

$$\Delta F^{\text{calc}}_{\text{iso}\to\text{nem}} = F_{\text{solv,nem}} - F_{\text{solv,iso}}, \tag{7}$$

as this determines the thermodynamic preference of the complex to reside in one phase or the other at equilibrium. Figure 2 plots the difference between the integrands in Eq. 6 for the isotropic and nematic phase, for two simulation trajectories at each λ. Numerical integration of the results in Figure 2 yields an estimate for $\Delta F^{\text{calc}}_{\text{iso}\to\text{nem}}$. Using the trapezoid rule for integration, we compute a value of $\Delta F^{\text{calc}}_{\text{iso}\to\text{nem}} = 334.4 k_B T_{\text{real}}$ and $329.5 k_B T_{\text{real}}$ (for simulation replicas 1 and 2 respectively).

We must then subtract the potential energy contribution $U_{\text{field,nem}}$ arising from interaction between the mesogens and the external electric field in the nematic case; this is because the electric field serves only to promote alignment of the mesogens, but besides this does no work on the system. This contribution can be easily extracted from our simulations via the average nematic order parameter $\langle P_2 \rangle$:

$$U_{\text{field,nem}} = -|q|(b-1)EN_{\text{rods}}L_{\text{bond}}\sqrt{\frac{2\langle P_2\rangle + 1}{3}}. \tag{8}$$

Here, E is the electric field strength (as used in Eq. 4), N_{rods} is the number of mesogens in the system, L_{bond} is the length of the bond connecting two beads in a mesogen, and b is the number of beads per mesogen. In the present case we have $N_{\text{rods}} = 10000$, $L_{\text{bond}} = 0.5\mathcal{D}$, and $b = 4$ and $|q|$ is unity. The average nematic order is computed by

$$\langle P_2 \rangle = \frac{1}{N_{\text{rods}}} \sum_{i=1}^{N_{\text{rods}}} \frac{1}{2} \left[3 \left(\frac{\mathbf{r}_i \cdot \mathbf{u}_x}{||\mathbf{r}||} \right)^2 - 1 \right], \tag{9}$$

where $\mathbf{r}_i$ is the orientation vector for mesogen i, and $\mathbf{u}_x$ is the unit vector along x. The true free energy difference of solvation is then

$$\Delta F_{\text{iso}\to\text{nem}} = \Delta F^{\text{calc}}_{\text{iso}\to\text{nem}} - U_{\text{field,nem}}. \tag{10}$$

In simulation, we find $U_{\text{field,nem}} = 94.1 k_B T_{\text{real}}$ and $91.1 k_B T_{\text{real}}$ for replicas 1 and 2 respectively. Removing this contribution yields $\Delta F_{\text{iso}\to\text{nem}} = 240.3 k_B T_{\text{real}}$ and $238.0 k_B T_{\text{real}}$. The value is large and positive, indicating that it is strongly *unfavorable* for the complex to reside in the nematic phase, relative to the isotropic one. (In text, we refer to $\Delta F_{\text{iso}\to\text{nem}}$ as "$\Delta\Delta G$".)

To map to experiment, it is convenient to represent the free energy difference in terms of the occupied volume of the complex. Here, the occupied volume of the complex includes the space occupied by the nanoparticle core, as well as the shell of space occupied by the stiff ligands: $V_{\text{occ}} = 4\pi[R_{\text{NP}} + bL_{\text{bond}}]^3/3 = 1437\mathcal{D}^3$ (where the excluding radius $R_{\text{NP}} + bL_{\text{bond}} = 7\mathcal{D}$). Dividing the average difference in solvation free energy, $\approx 240 k_B T_{\text{real}}$, by this volume yields $0.166 k_B T_{\text{real}}/\mathcal{D}^3$.

In experiment, the excluding radius of the NP complex is roughly 4.5nm (the core is about $2-3$nm in radius, and the ligands extend about $1-2$nm off the core surface). This allows us to define a length scale mapping between the molecular dynamics model and experiment, namely 4.5nm $\to 7\mathcal{D}$, from which we can predict the solvation free energy difference per unit volume (in nm^3) in experiment:

$$\frac{\Delta F_{\text{iso}\to\text{nem}}}{\text{nm}^3} = \left(\frac{\Delta F_{\text{iso}\to\text{nem}}}{V_{\text{occ}}}\right) * \left(\frac{7\mathcal{D}}{4.5\text{nm}}\right)^3 \approx 0.62 \left(\frac{k_B T_{\text{real}}}{\text{nm}^3}\right). \tag{11}$$

This effectively pins the length-scale of the molecular dynamics model based on size of the nanoparticle complex in experiment.

The difference in solvation free energy can then be written in terms of its contributing factors:

$$\Delta F_{\text{iso}\to\text{nem}} = \Delta U_{\text{inter}} + \Delta U_{\text{intra}} + \Delta U_{\text{solvent}} - T_{\text{real}}(\Delta S_{\text{intra}} + \Delta S_{\text{solvent}}) \tag{12}$$

where ΔU_{inter} is the difference in intermolecular Lennard-Jones potential energy between the complex and solvent, ΔU_{intra} is the difference in *intramolecular* interaction energy within the complex itself (including Lennard-Jones, bond, and angle contributions), and $\Delta U_{\text{solvent}}$ is the difference in potential energy of the solvent (again including the latter three pieces). The last two terms are the entropy change of the complex and solvent, respectively.

The potential energy differences can be easily extracted from simulation: we find the following average values (in units of $k_B T_{\text{real}}$):

- $\Delta U_{\text{inter}} = -0.5$
- $\Delta U_{\text{intra}} = 56.1$
- $\Delta U_{\text{solvent}} = -72.5$

The intermolecular energy change ΔU_{inter} is very small, as on average the complex has the same degree of contact with the solvent in both phases. With these quantities known, we can solve for the entropy change terms: $T_{\text{real}}(\Delta S_{\text{intra}} + \Delta S_{\text{solvent}}) = -348.9 k_B T_{\text{real}}$, or

$$\frac{T_{\text{real}}(\Delta S_{\text{intra}} + \Delta S_{\text{solvent}})}{\text{nm}^3} = -0.9 \left(\frac{k_B T_{\text{real}}}{\text{nm}^3}\right) \tag{13}$$

The NP complex itself is rigid, differing little in configurational behaviour in the isotropic and nematic phase. We therefore expect the $T\Delta(\Delta S_{\text{intra}})$ contribution to be minimal. Thus, the full entropic penalty of $-0.9 k_B T/\text{nm}^3$ is largely due to the solvent contribution $\Delta(\Delta S_{\text{solvent}})$. This strong penalty arises from how the NP complex distorts the surrounding mesogens from their preferred nematic orientation. While these mesogens appear to be "more disordered", they actually pack less efficiently in the distorted state, leading to overall *fewer* configurational options relative to when the NP complex is not present. By tuning the miscibility of the ligands with the LC, the relative magnitude of this entropy penalty may be offset so that the NP complexes have a stronger or weaker preference for solvating in the nematic phase.

Phase Field Simulation

In order to capture the phenomenology of microscale patterning of nanoscale building blocks by phase separation, we modelled the morphological evolution of the phase transition and NP segregation with an Allen-Cahn equation for a coarse-grained directional order-parameter field $\eta(\vec{x}, t)$ and a Cahn-Hilliard equation for mesogen/NP composition $c(\vec{x}, t)$. In this model, $\eta = \hat{n}(\vec{x}) \cdot \hat{s} = 1$ represents complete directional order, i.e. all mesogen director fields $\hat{n}(\vec{x})$ are by the aligned substrate into the direction $\hat{s}$. Where $\eta = 0$, coarse-grained LC alignment to the substrate is absent; for the composition $c = 1$, the system is pure mesogen and $c = 0$ indicates pure NP. The results of an example simulation appears in Fig. 4.

The simulation illustrates the morphological evolution of system with initially no long-range directional-order and a uniform composition of NPs as it decomposes into: 1) a NP-enriched phase with small substrate-induced long-range directional order; 2) a phase from which NPs are depleted with long-range directional order. The first modeling hypothesis is that the substrate induces a uniform preferred direction within the nematic phase in which η tends to 1. In the absence of a substrate, $\eta(x)$ tends to zero but there can be microdomains where the director field has short range correlations. The second modeling hypothesis is that nanoparticles diminish the tendency for long-range directional order in their vicinity. Thus, the NPs (i.e. where c is small) diminish the substrate's induced-direction effect. A phenomenological free energy couples $\eta(\vec{x}, t)$ to $c(\vec{x}, t)$ and is motivated by an equilibrium between 1) the low enthalpy and low entropy NP-depleted nematic phase that is aligned in the presence of a

substrate; 2) the moderate enthalpy and high entropy NP-rich phase with no long-range directional order. This free energy is illustrated by the contour plot Fig. 4(g) is a model for these two coupled hypotheses.

The locations of the maxima, minima, and saddle points of $\Delta g(\eta, c)$ were chosen to be consistent with the hypotheses presented above as described in Fig. 4's caption.

The free energy's local minimum in the upper-right corner in Fig. 4(g). ($c \approx 1$ and $\eta \approx 1$) represents NP-depleted material that has long-range substrate-induced uniform directionality; the lower-left local minimum represents material where the presence of many NPs diminish the substrate's long-range ordering effect. The equilibrium compositions and their η would be obtained by a common tangent construction in the (conserved) composition axes–that is, a common tangent construction of the free-energy surface $\Delta g(c, \eta)$ projection onto a constant η-plane[5].

The microstructural evolution behavior is relatively insensitive to small changes in the free-energy function. The initial decomposition followed by break-up would occur for any composition–order system where the initial state lies in an unstable region. The compositions and long-range-order of the final homogeneous phases depend on the location of the two local minima and the common-tangent construction. The interfacial energy is determined by an integral ($\int_{c(s_1)}^{c(s_2)} \Delta g(c(s), \eta(s) + k_c(\nabla c(\vec{x}(s)) \cdot \nabla c(\vec{x}(s)) + k_\eta(\nabla \eta(\vec{x}(s)) \cdot \nabla \eta(\vec{x}(s)\ ds$) where $\vec{x}(s)$ is the spatial path between two equilibrium homogeneous phases.

The morphological evolution was simulated with a phase-field model. Allen-Cahn dynamics apply to the non-conserved directional-order parameter $\eta(\vec{x}, t)$. Cahn-Hilliard dynamics apply to $c(\vec{x}, t)$ and require that spatial variations in $c(\vec{x})$ preserve system's average composition. The coupling of $c(\vec{x})$ and $\eta(\vec{x})$ occurs via the phenomenological free-energy function, $\Delta g(c, \eta)$ such as that illustrated in Fig. 4(g).

The system will spontaneously decompose into NP-rich and NP-depleted regions at any $\Delta g(\eta, c)$ where at least one of the principle curvatures of the surface $\Delta g(c, \eta)$ is negative–this satisfies the conditions of spinodal decomposition. The "direction" of decomposition ($\frac{\partial c}{\partial t}/\frac{\partial \eta}{\partial t}$) is restricted to those directions that have a negative curvature; the specific direction depends on that curvature and the ratio of the two time constants (mobilities). If LC directional-ordering is fast, then the developing equal-time curves in Fig. 4 will have large vertical separations compared to the difference of the c-values at their end-points. The morphological evolution has two stages: initially, the NPs segregate into spinodal laminae with a characteristic wavelength and is dominated by reduction in the bulk free energy; subsequently, the laminae break up and coarsen, resulting in isolated NP-rich regions enveloped into a NP-depleted and long-range directionally ordered phase. The second stage is dominated by reduction in interfacial energy.

The simulations illustrated in Fig. 4 can be physically interpreted as follows. At $t = 0$, a system with no long-range substrate-induced directional-order ($\eta \approx 0$) system initially adopts long-range order that is driven by an effective "ordering" field generated by the substrate. As directional-order increases, concomitant NP-segregation and loss of directional order develops (the pink region in Fig. 4(a) indicates directional order and is scaled to accentuate $0 < \eta 0.1$; grayscale indicates the composition variation observed in optical microscopy) The regions that have depleted NPs have a larger driving force to increase η that enhances phase the η-separation in the c–η plane. This initial decomposition can be observed in Fig. 4(a-b) and has the morphology of a spinodal decomposition. The late-stage system morphology is composed of NP-depleted/aligned nematic phase (white in Fig. 4(a-f)) that envelopes a small amount of nearly random nematic NP-rich phase (i.e., $\eta \approx 0.2$). The final state does not have the bi-phase percolating (e.g., "brain-like") morphology that is commonly associated with a $X_A = X_B = 1/2$ spinodal composition. Instead, the initial laminar fluctuations break the minor phase into isolated regions–this produces a final structure that is more commonly associated with nucleation and growth; but in this case, the structure is initiated by a decomposition reaction.

The colored curves in Fig. 4(g) illustrate time-dependent co-variation of composition and order across the interface. They are computed by fitting the collective pixels' c and η (i.e., the black dots) to a second-order curve. The homogeneous phases occupy the most of the microstructure and are representented by the end-points the developing curves. The salient black points in Fig. 4(g) derive from the agglomerate microstructure at a particular time–while the points produce the most notable aspect when projected onto the free-energy plane, most of the microstructure's volume is represented by a larger number of points superimposed at the interfacial curve's endpoints.

The developing interfacial curves in Fig. 4(g) should evolve toward a fixed curve that minimizes the surface tension in the system. Presumably this minimizing curve should pass near the saddle point of $\Delta g(\eta, c)$ but is also influenced by the square gradient coefficients in the free energy functional. The full analysis of the direction of initial composition and the equilibrium interface is beyond the scope of this paper. The initial decomposition produces a large amount of interface. At small times, points in Fig. 4 come from a multitude of interfaces. At larger times, the system coarsens and removes many of the interfaces. It is possible to construct a free-energy function for which the final microstructure is LC with solubilized NPs–and which undergoes an initial decomposition reaction. In other words, if the free energy function has regions of negative curvature for small values of η, it is possible for the system to undergo a transient spinodal decomposition. When the compositions reach the spinodal boundary, those compositions catalyze direct growth like a super-critical nucleus.

EXPERIMENTAL RESULTS

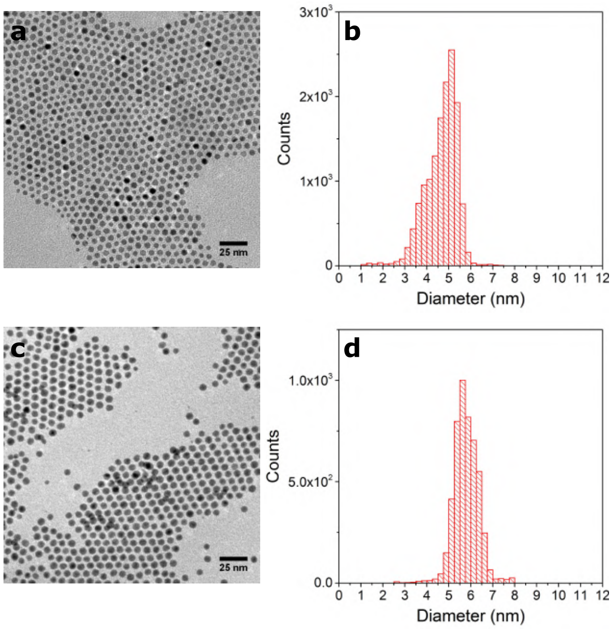

Supplementary Figure S 3 | Electron microscopy of gold nanoparticles. **a**, Representative TEM image of the AuNPs used in this study. **b**, Size histogram of the analysed AuNPs, with a total count of 14,300 and a mean size of 4.7 ± 0.7 nm. **c**, Representative TEM image of the AuNPs used in the self-assembly study to investigate size effects. **d**, Size histogram of the AuNPs with a total count of 5,100 and a mean size of 5.9 ± 0.9 nm.

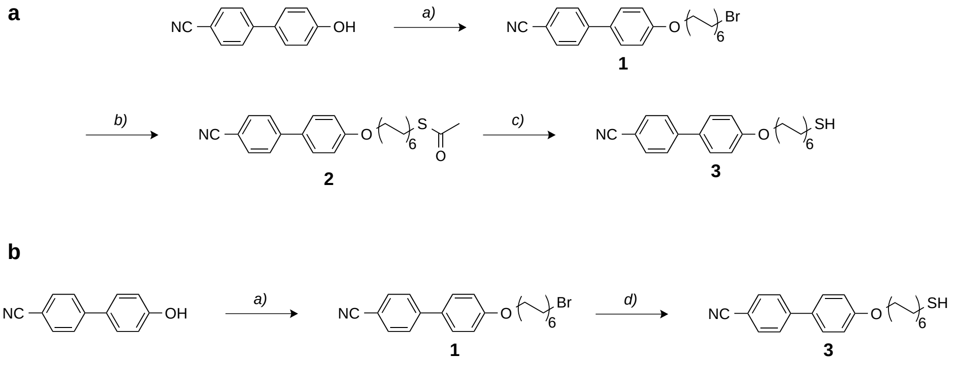

Supplementary Figure S 4 | Ligand synthesis. The 4'-(12-mercaptododecyloxy)-4-biphenylcarbonitrile **3** was prepared from commercially available starting material in few synthetic steps via (**a**) the thioacetate **2** or (**b**) directly from the bromine derivative **1** following a literature procedure from Milette and coworkers.[6] *Reagents and Conditions: a)* 1,12-dibromododecane, K_2CO_3, acetone, 88%; *b)* KSAc, DMF, 94%; *c)* AcCl, DCM, MeOH, 46%; *d)* hexamethyldisilathiane, TBAF, THF, 74%. The detailed experimental procedures are described in the Methods section and the NMR spectra of compounds **1**-**3** are depicted in the Supplementary Figures **S9**-**S11**, respectively.

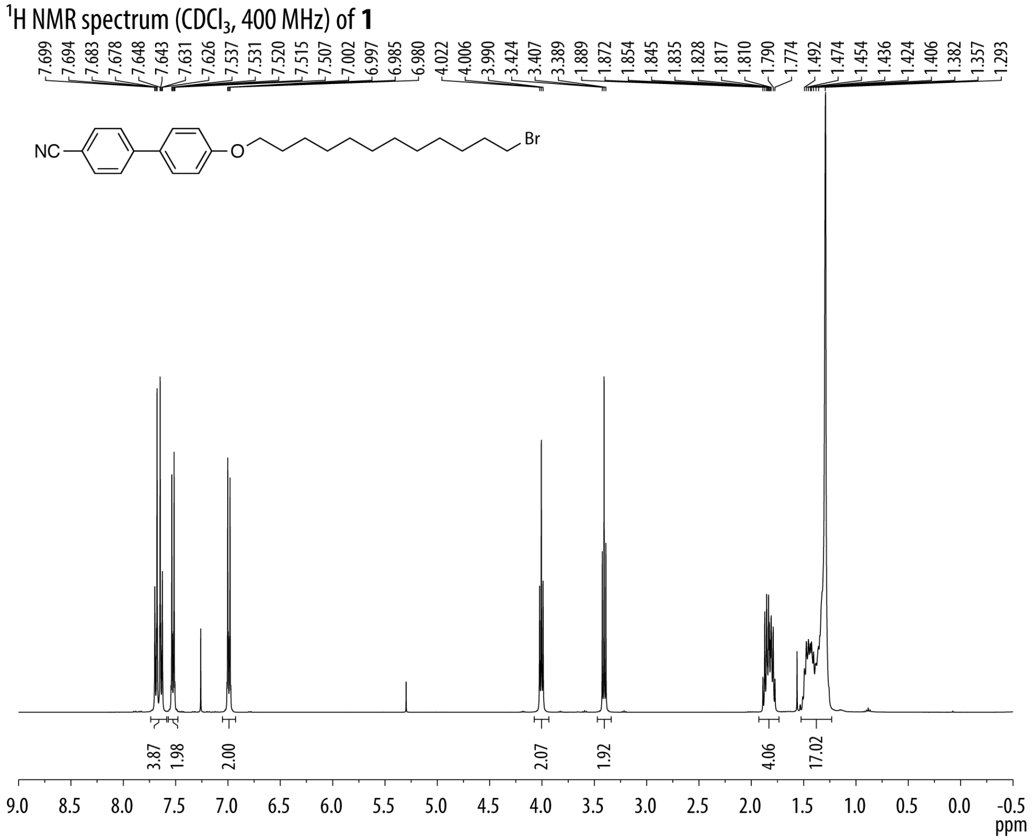

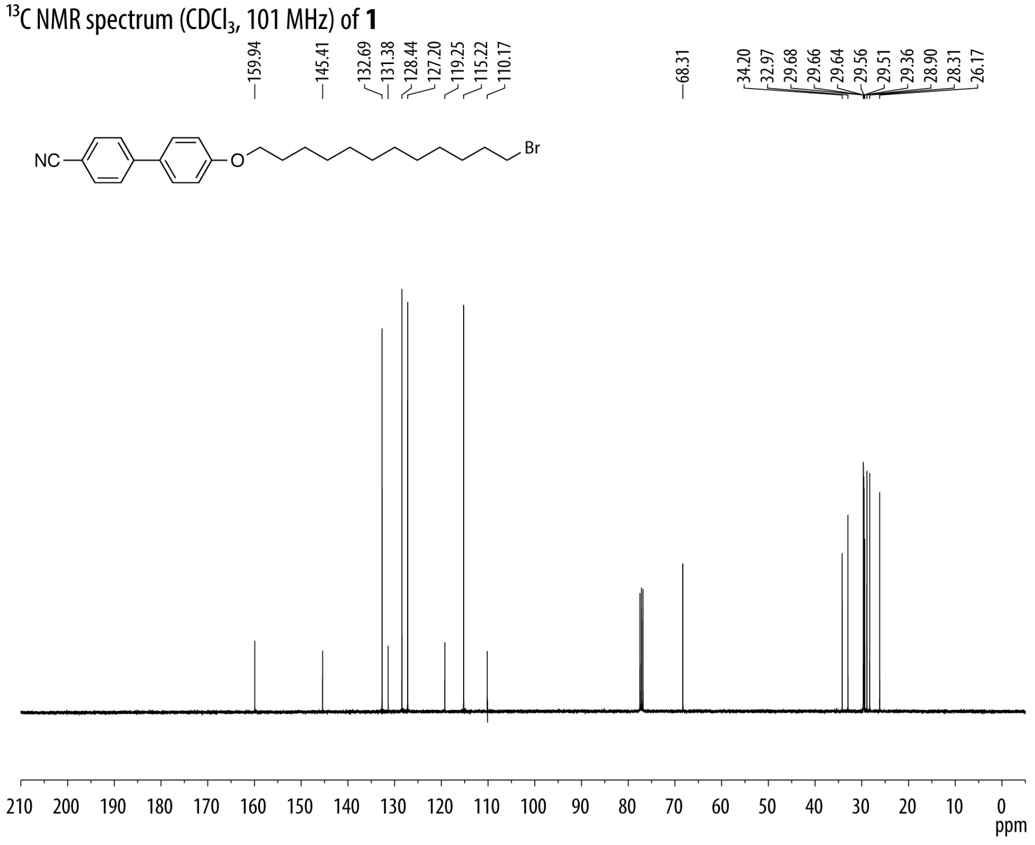

Supplementary Figure S5 | ^{1}H and ^{13}C NMR spectra of 4'-(12-Bromododecyloxy)-4-biphenylcarbonitrile 1.

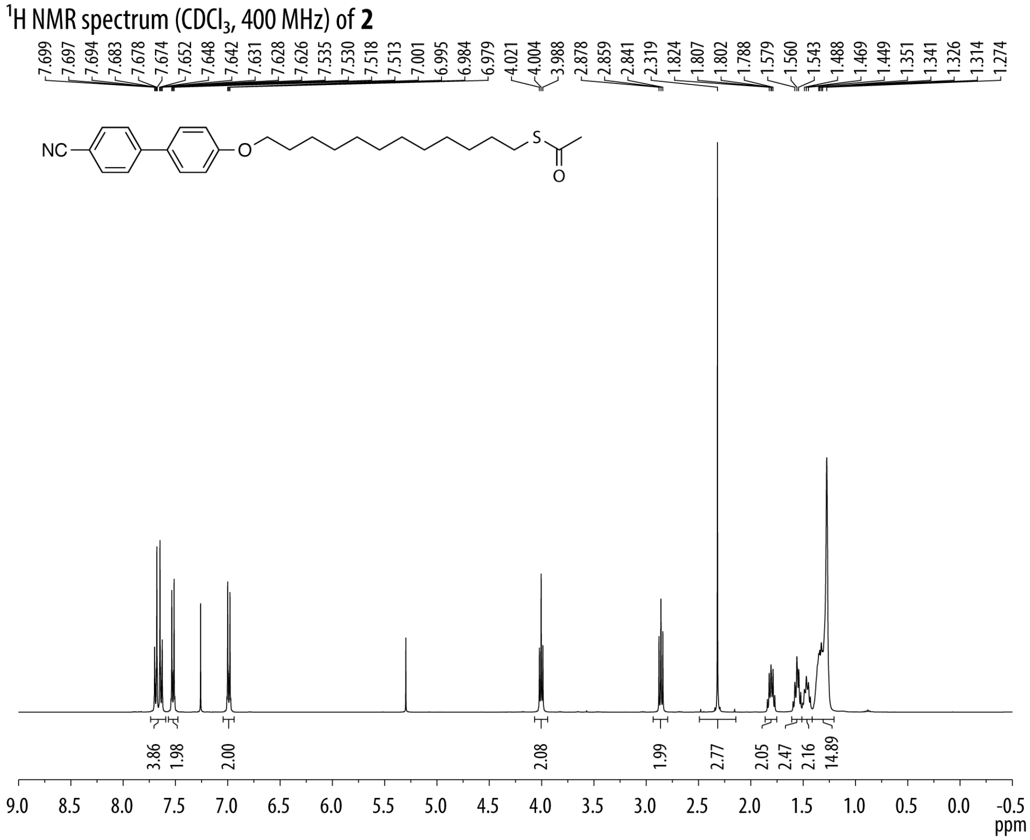

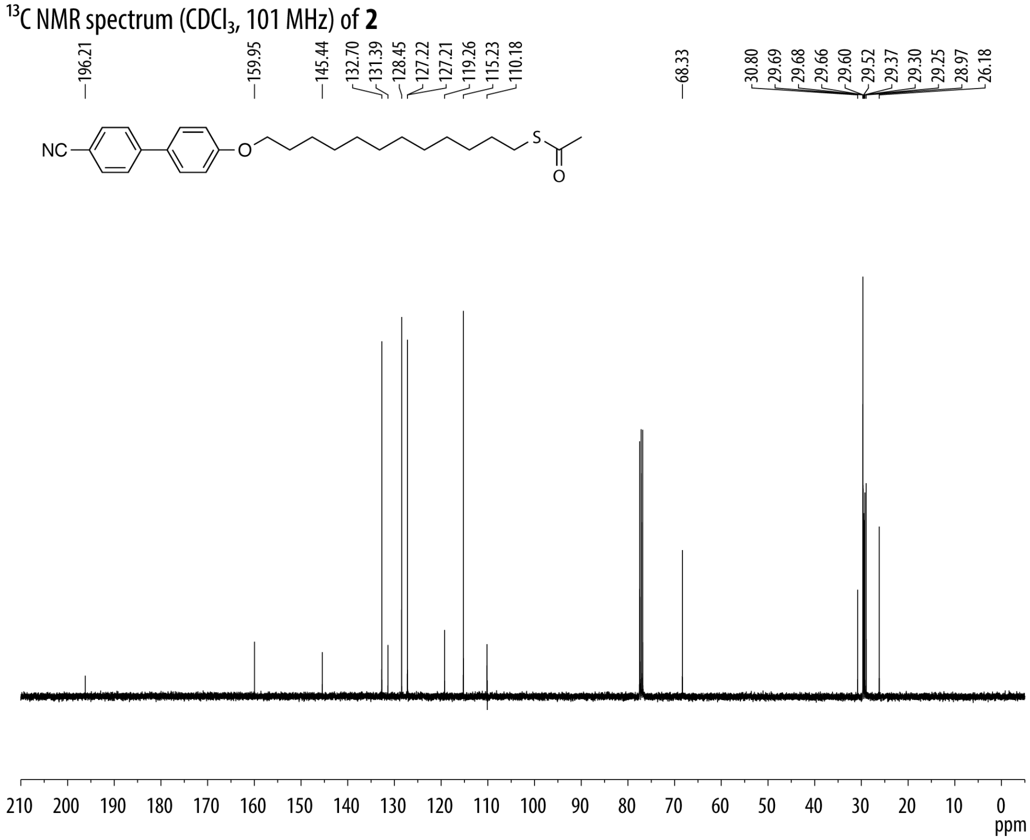

Supplementary Figure S 6 | ^{1}H and ^{13}C NMR spectra of *S*-(12-(4'-(4-Biphenylcarbonitrile)oxy)dodecyl) ethanethioate 2.

^1H NMR spectrum ($CDCl_3$, 400 MHz) of **3**

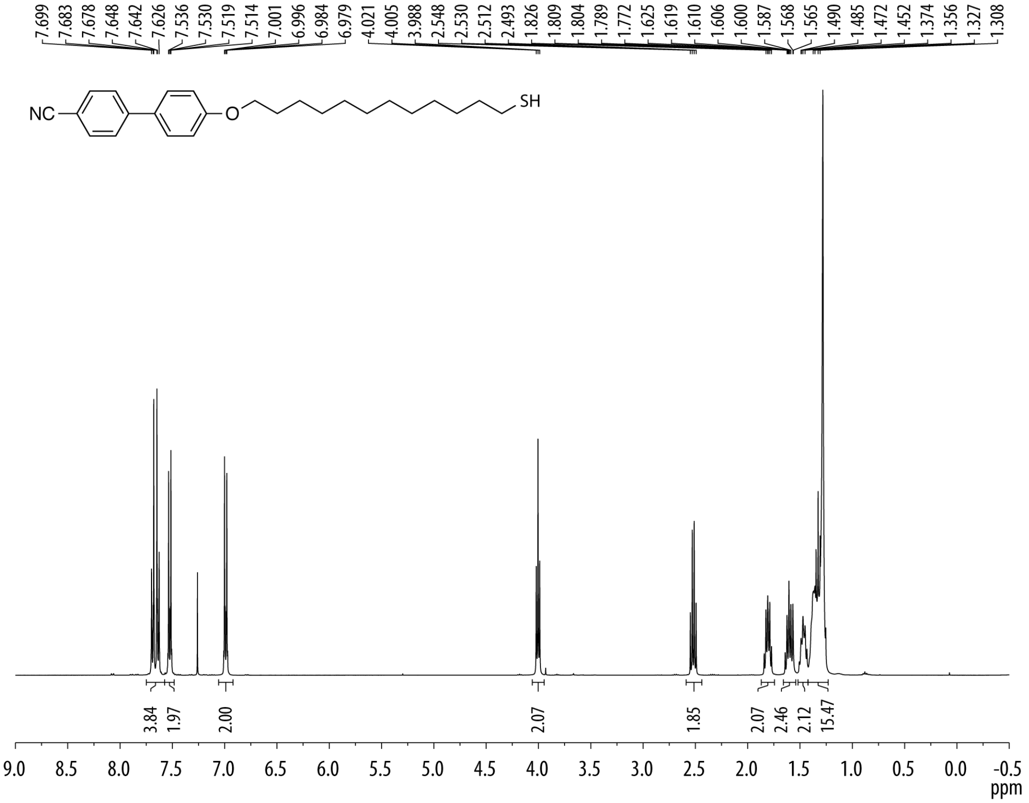

^{13}C NMR spectrum ($CDCl_3$, 101 MHz) of **3**

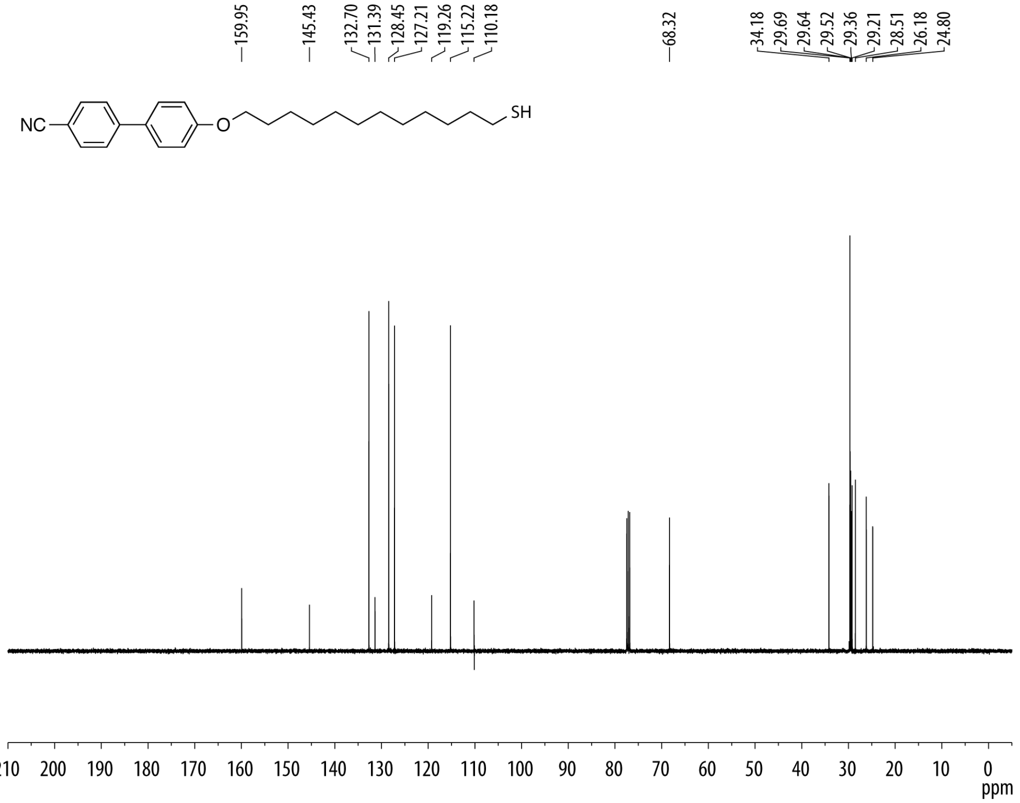

Supplementary Figure S7 | ^{1}**H and** ^{13}**C NMR spectra of 4'-(12-Mercaptododecyloxy)-4-biphenylcarbonitrile 3.**

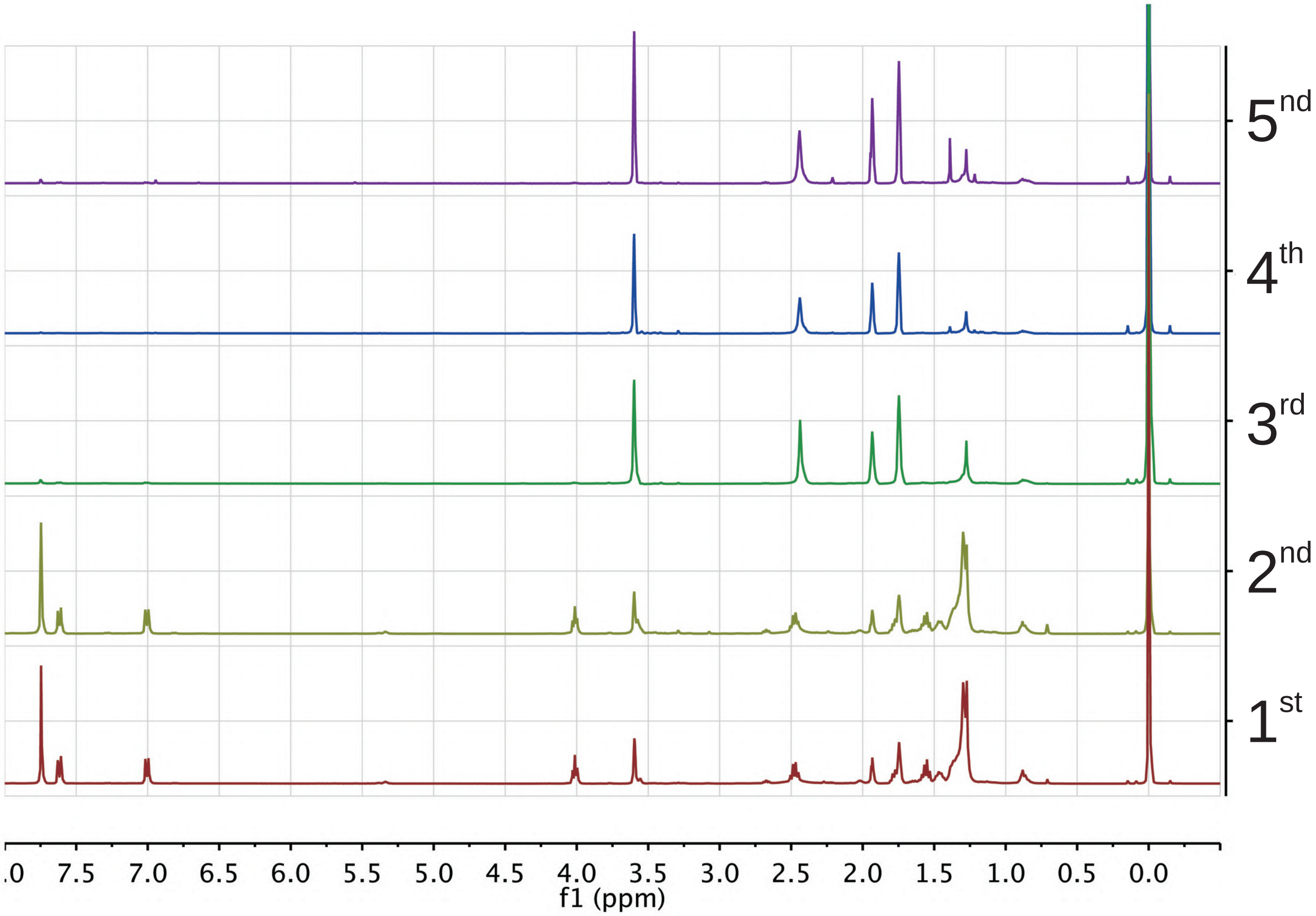

Supplementary Figure S 8 | Nanoparticle cleaning. NMR spectra of supernatant in five consecutive cleaning cycles. In step 1-3, NPs were dispersed in a 10/90 vol% mixture of DCM and acetone and precipitated by ultracentrifugation (32,000 rpm, 1 h). In step 4-5, a mixture of 10/10/80 vol% THF, acetonitrile and acetone was used. The supernatant was subsequently dried and the solid residuals redissolved in a 50/50 vol% mixture of deuterated THF and acetonitrile before NMR analysis.

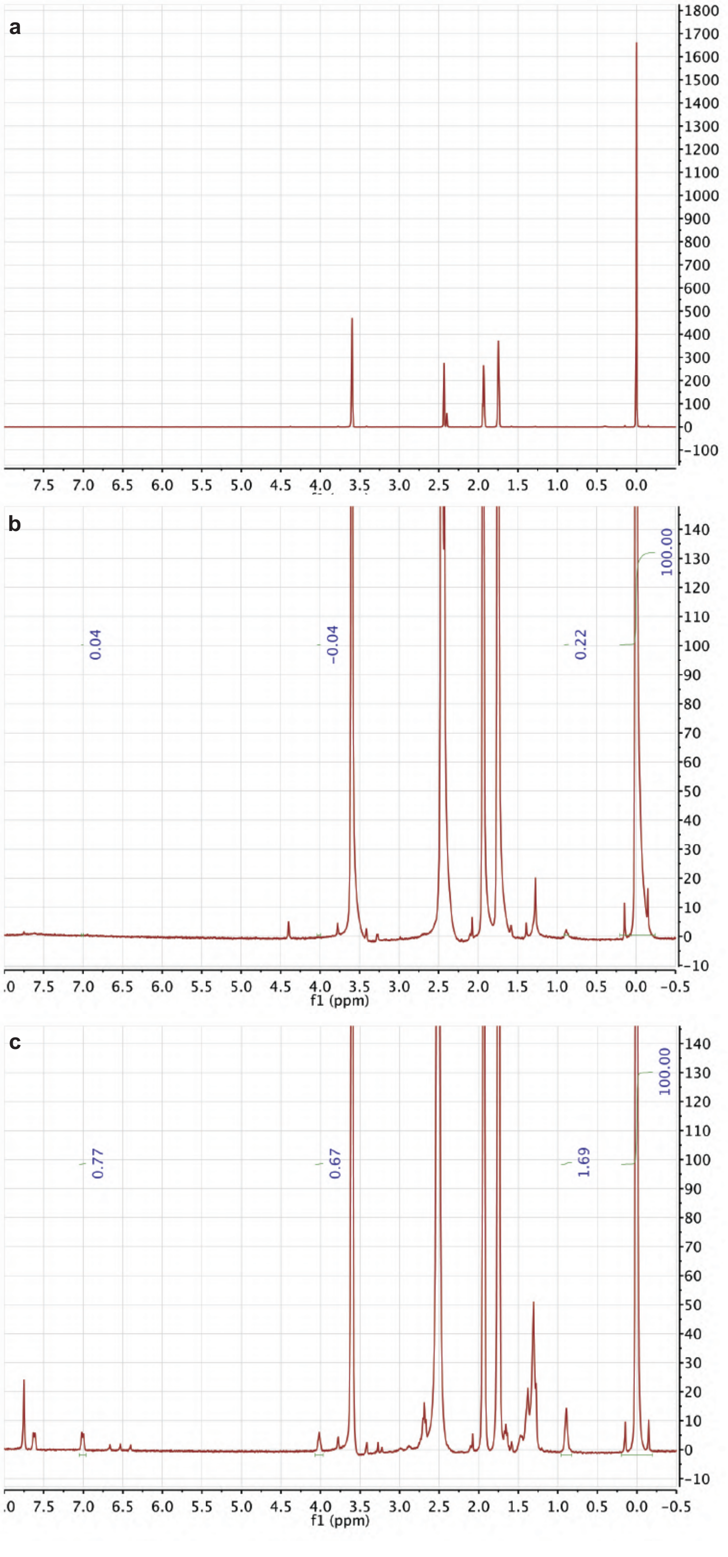

Supplementary Figure S9 | NMR analysis of NPs. NMR was first conducted of the thiol-protected NP solution during the various stages of NP clean-up to verify the absence of unbound ligands.[7] Subsequently the Au core of the NPs was etched to release the surface bound ligands for quantitative analysis. **a** NMR spectra of the solvent mixture only (50/50 vol% deuterated THF and acetonitrile). **b** NMR spectra of NPs after 5 cleaning cycles. **c** NMR spectra after the addition of iodine to etch the gold core and release the surface-bound ligands. Analysis of the peak area at 7.03 ppm (MDD-CBO - 2H), 4.01 ppm (MDD-CBO - 2H) and 0.85 ppm (HT - 3H) for multiple samples is consistent with a ligand ratio of $61/39 \pm 2$ HT/MDD-CBO. In a separate batch, a ratio of $61/39 \pm 3$ HT/MDD-CBO was determined. Please note that a feed ratio of 60/40 was applied in the synthesis.

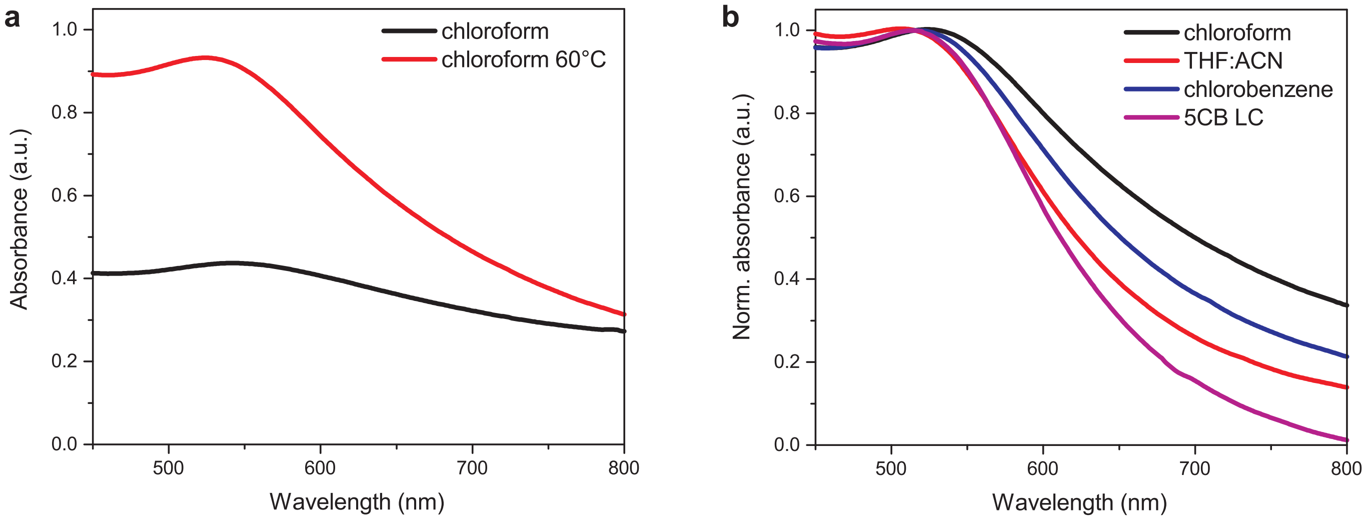

Supplementary Figure S 10 | Uv-vis spectroscopy of NPs in various solvents. **a** Absorbance of NPs in chloroform before and after exposure to an overnight temperature protocol at 60°C . **b** Absorbance of NPs in a variety of solvents after overnight exposure to 60°C . Note that for comparison the absorbance value was normalised at $\lambda = 515$ nm. The 5CB liquid crystal was kept in the isotropic phase.

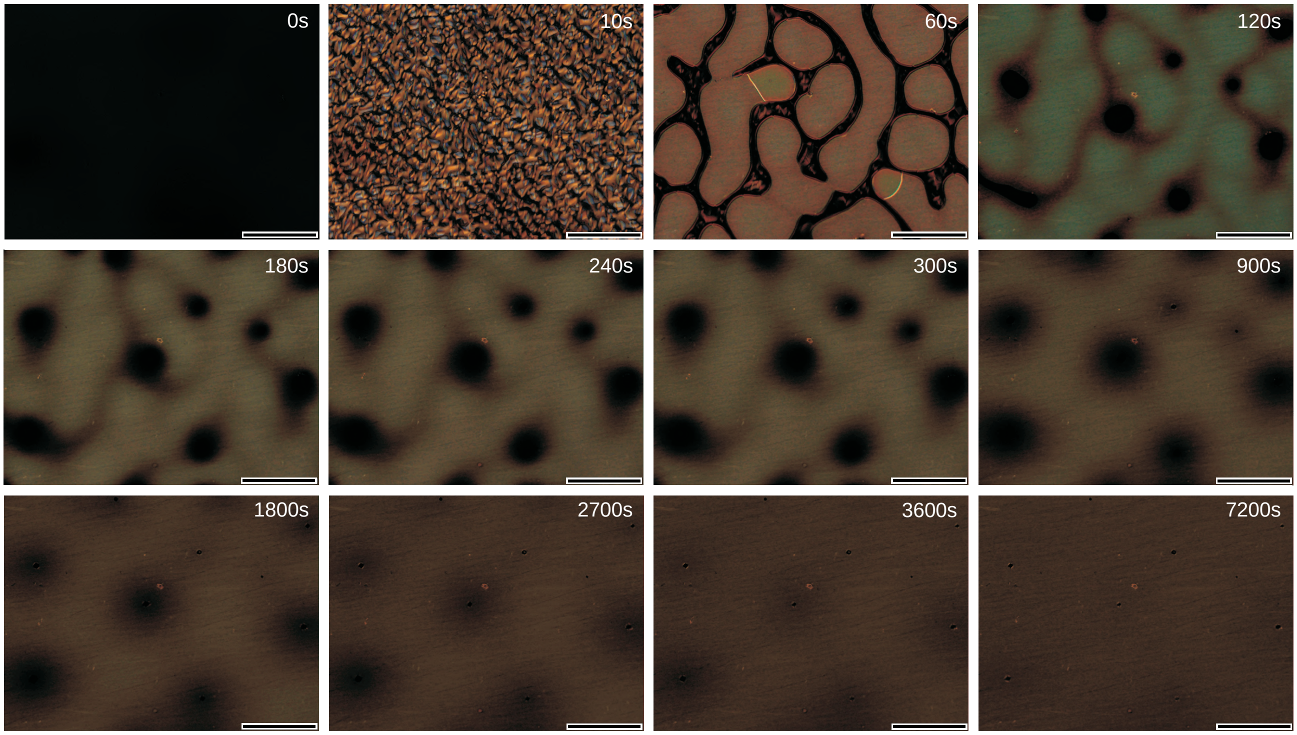

Supplementary Figure S 11 | LC isotropic-nematic phase transition under cross polarisation. Kinetic series of microscopy images under cross-polarisation upon cooling of the of LC-NP composite (5w% NP) from 40.0 °C to 28.0 °C (1°C/min), with image acquisition commencing at 34.3 °C (10x objective). The scale bar represents 200 μm . Note that a video of this experiment is available for further illustration.

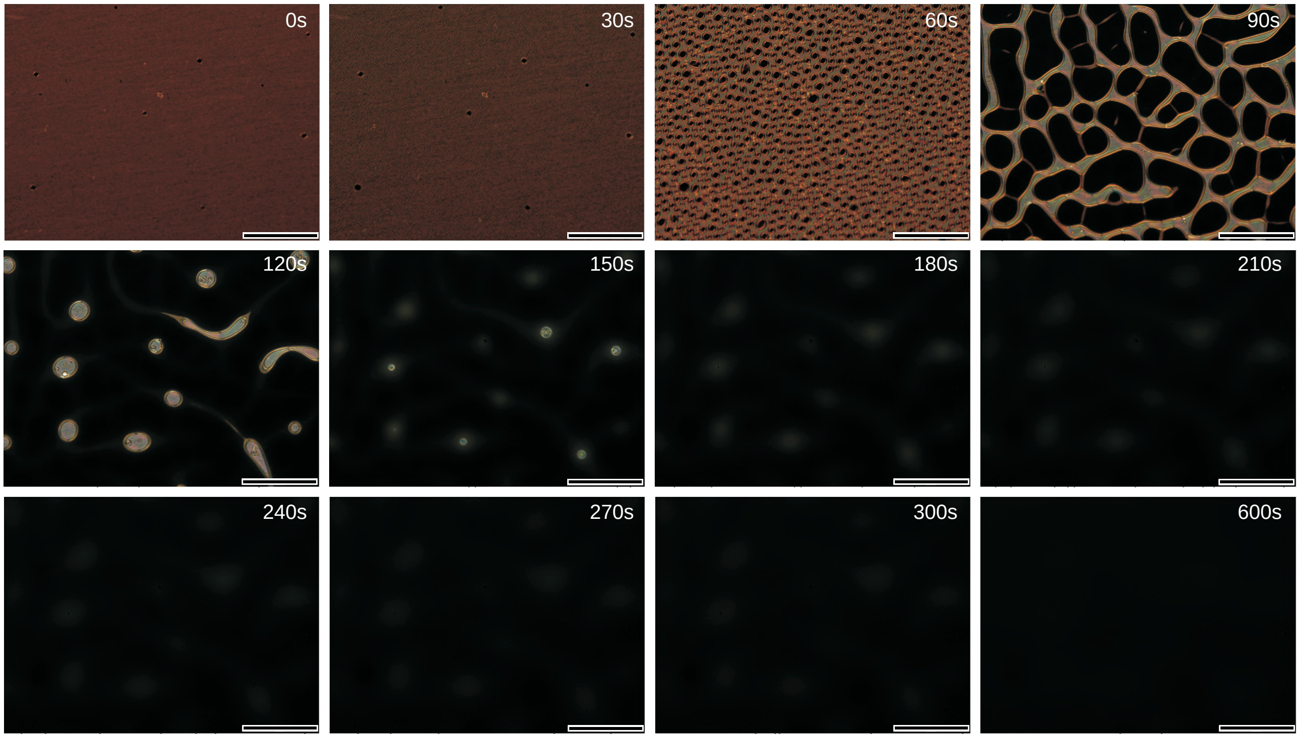

Supplementary Figure S 12 | LC nematic-isotropic phase transition under cross polarisation. Kinetic series of microscopy images under cross-polarisation upon heating of the of LC-NP composite (5w% NP) from 28.0 °C to 40.0 °C (1°C/min) with image acquisition commencing at 34.7°C . The scale bar represents 200 µm .

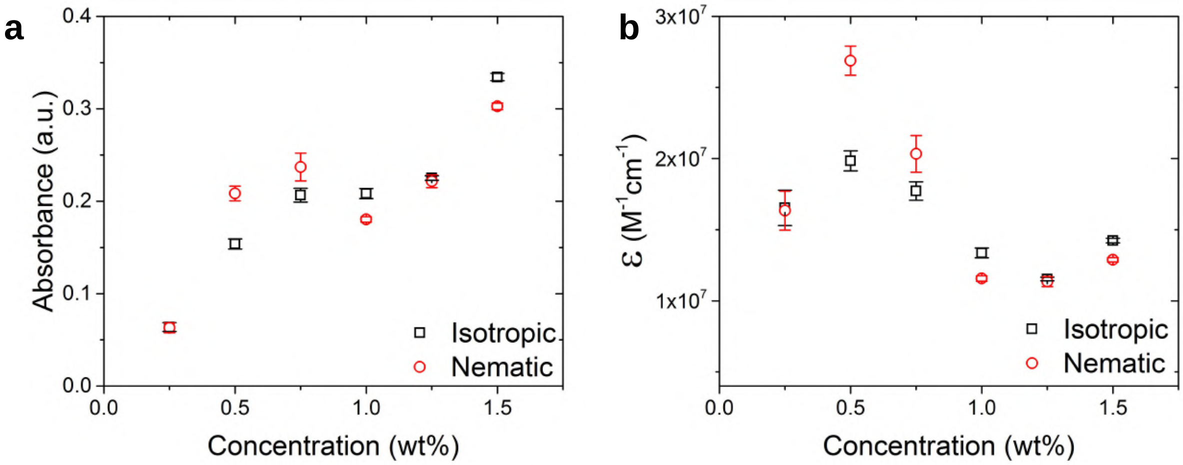

Supplementary Figure S13 | Molar attenuation coefficient (ϵ) of the AuNPs in the isotropic and nematic phases. The absorbance of the LC-NP composite was measured for the isotropic and nematic phases over a range of concentrations (**a**) and the molar attenuation coefficient calculated for each sample (**b**). In the observed range, an average molar attenuation coefficient of $(1.55 \pm 0.14) \times 10^7$ M^{-1}cm^{-1} was determined for the isotropic phase and $(1.66 \pm 0.44) \times 10^7$ M^{-1}cm^{-1} for the nematic phase. Note that the absorbance is largely influenced by fluctuations in the sample compartment thickness of the LC cell. A two sample T-Test confirmed that the molar attenuation coefficient is effectively the same for both phases

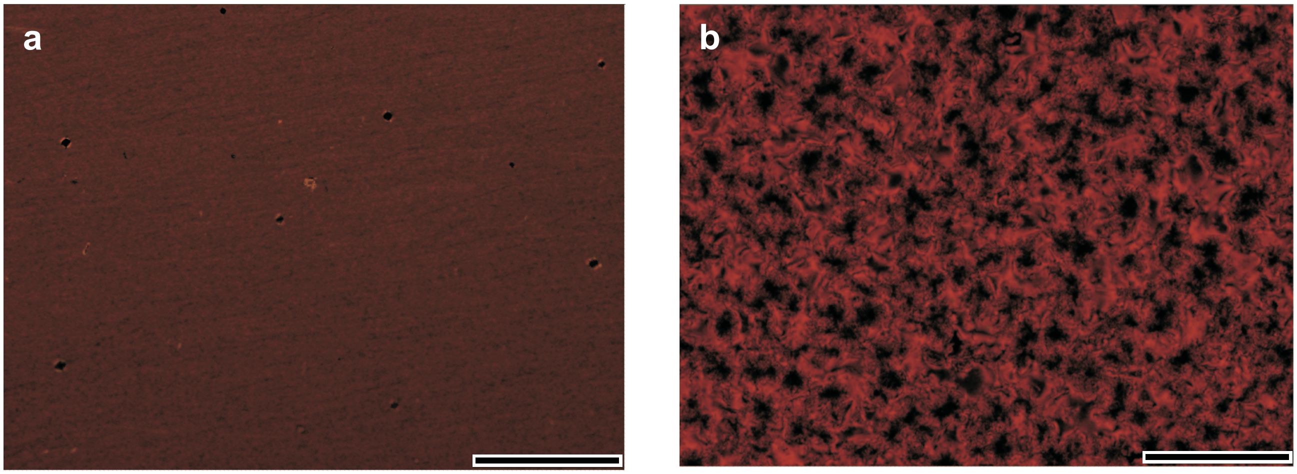

Supplementary Figure S14 | Effect of homogeneous surface alignment under cross polarisation. LC-NP composite (5w% NP) in the nematic phase (Figures SI13, SI15 at 0 s). **a** Commercial LC cell with polyimide layer and homogeneous surface alignment (20 µm spacing, Instec) **b** Custom-built glass sandwich without surface alignment ($\approx 20 - 25$ µm spacing). The scale bar represents 200 µm .

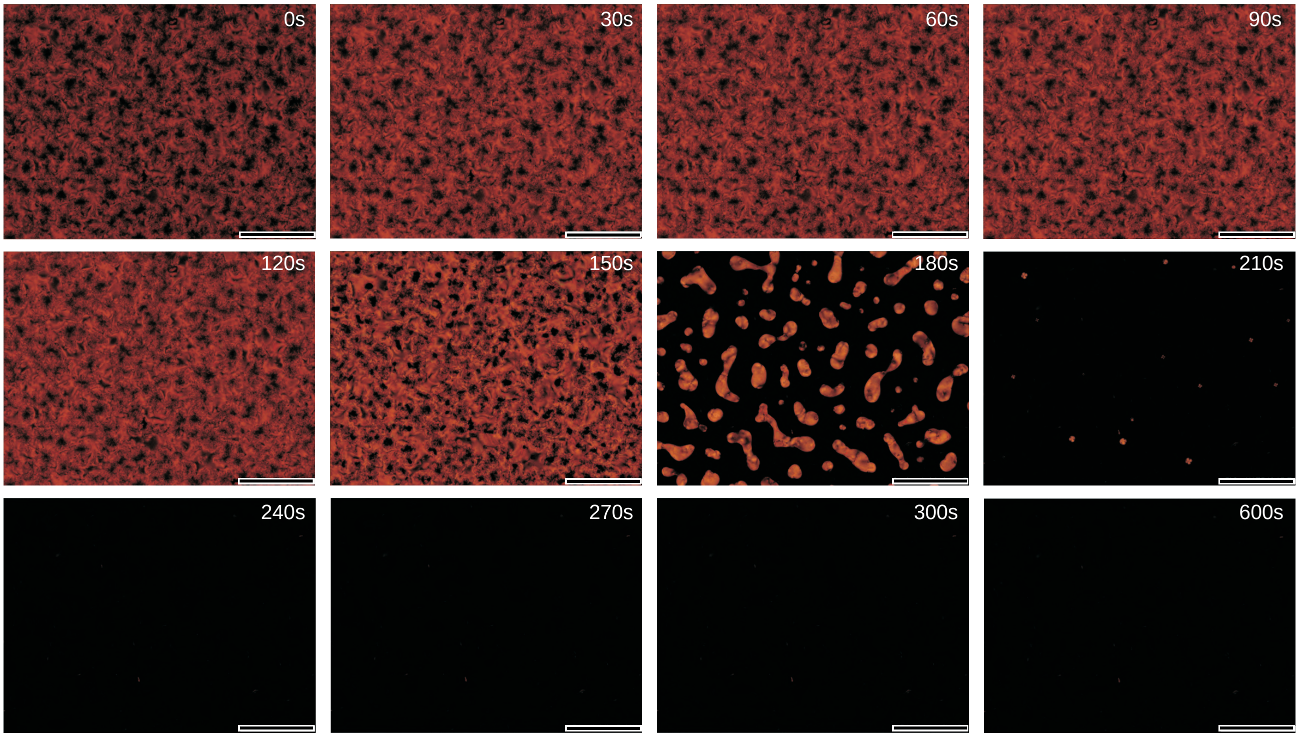

Supplementary Figure S15 | LC nematic-isotropic phase transition under cross polarisation without surface alignment. Kinetic series of microscopy images under cross-polarisation upon heating of the LC-NP composite (5w% NP) to 34.7°C (1°C/min). Here, a custom-built glass sandwich consisting of two microscope slides without surface alignment and a 25 µm thermoplastic sealant (DuPont Surlyn) was used. After processing, this will result in an effective spacing of $\approx 20 - 25$ µm . The scale bar represents 200 µm .

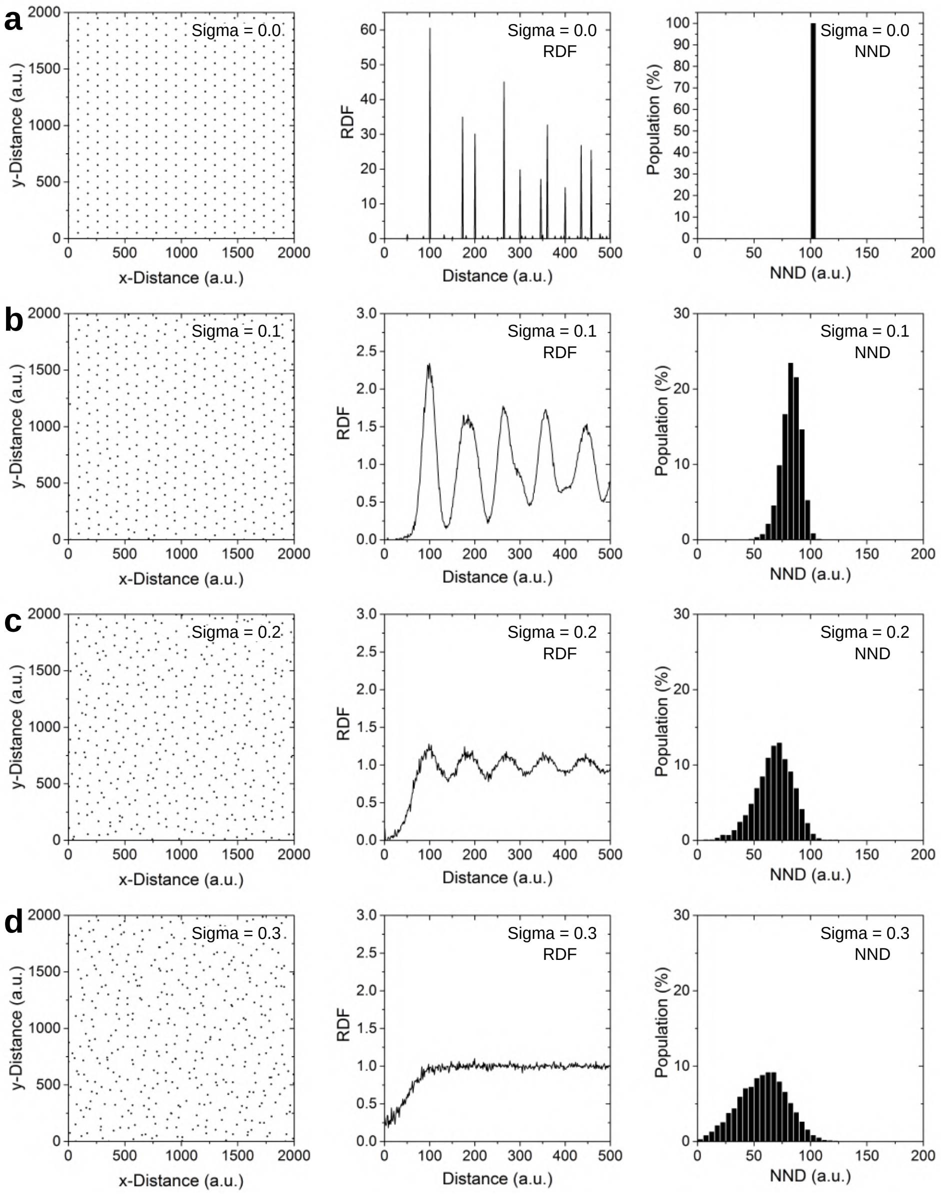

Supplementary Figure S16 | Nearest Neighbour Distance vs. Radial Distribution Function. A hexagonal array was created with a characteristic spacing of 100 a.u. Gaussian noise with a specified standard deviation (sigma) was then added. The array was then analysed with the radial distribution function (RDF) and the nearest neighbour distance function (NND) for a perfect hexagonal array (**a**) and a sigma of 0.1 (**b**) 0.2 (**c**) and 0.3 (**d**), respectively.

Besides the use of an RDF to evaluate ordering, we also considered the nearest neighbour distance (NND) as a viable method. To investigate the robustness of the analysis approach to small deviations of noise, a characteristic hexagonal pattern was created and gaussian noise with a set standard deviation (sigma) was added, see Supplementary Figure 16. The resulting test arrays were then analysed for RDF and NND. Once noise was added to the pattern, the corresponding NND shifted to lower values while the RDF maintained the true characteristic spacing. This is related to the underlying algorithm that calculates the inter-particle distance. The RDF function looks at all neighbouring particles in order to determine the characteristic spacing while the NND uses only the closest neighbouring particle which, due to the noise level, will lie closer than the true characteristic spacing. We therefore employed the RDF to evaluate ordering of the aggregates.

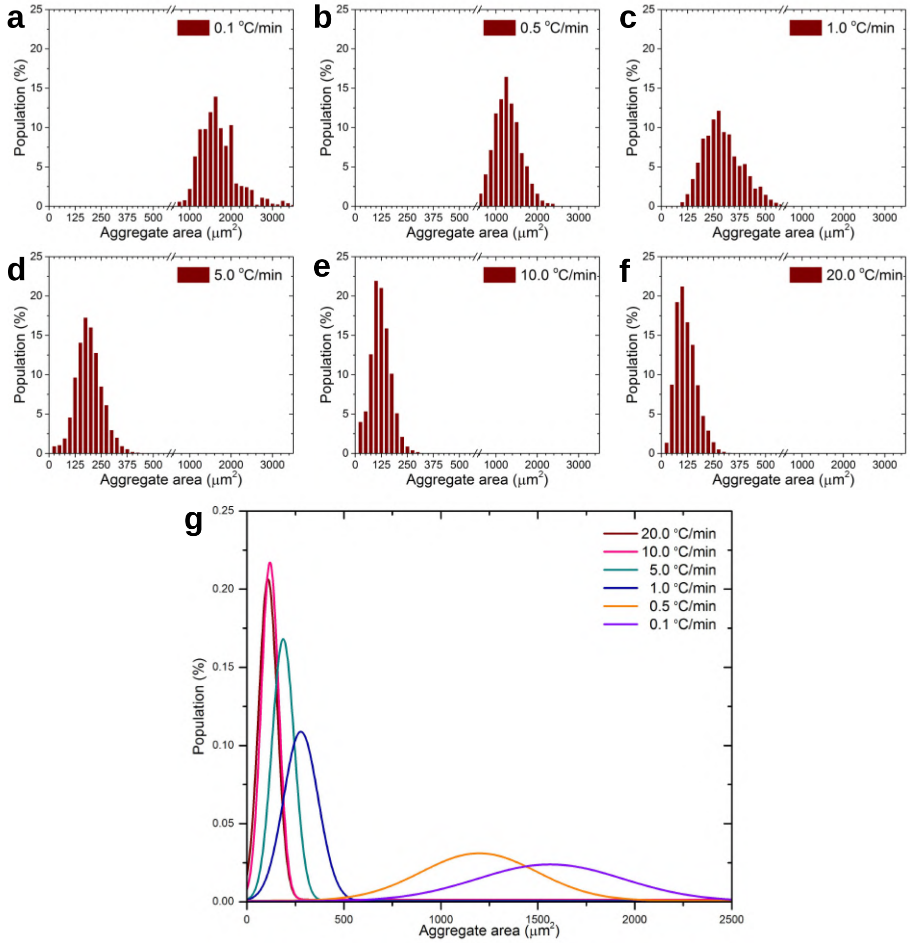

Supplementary Figure S17 | Effect of cooling rate on the aggregate size. The aggregate area was determined through image analysis of a composite bright field image of the active area of the LC cell. **a**-**f** The data was weighted by volume and plotted as a histogram. **g** A Gaussian function was fitted to the data sets.

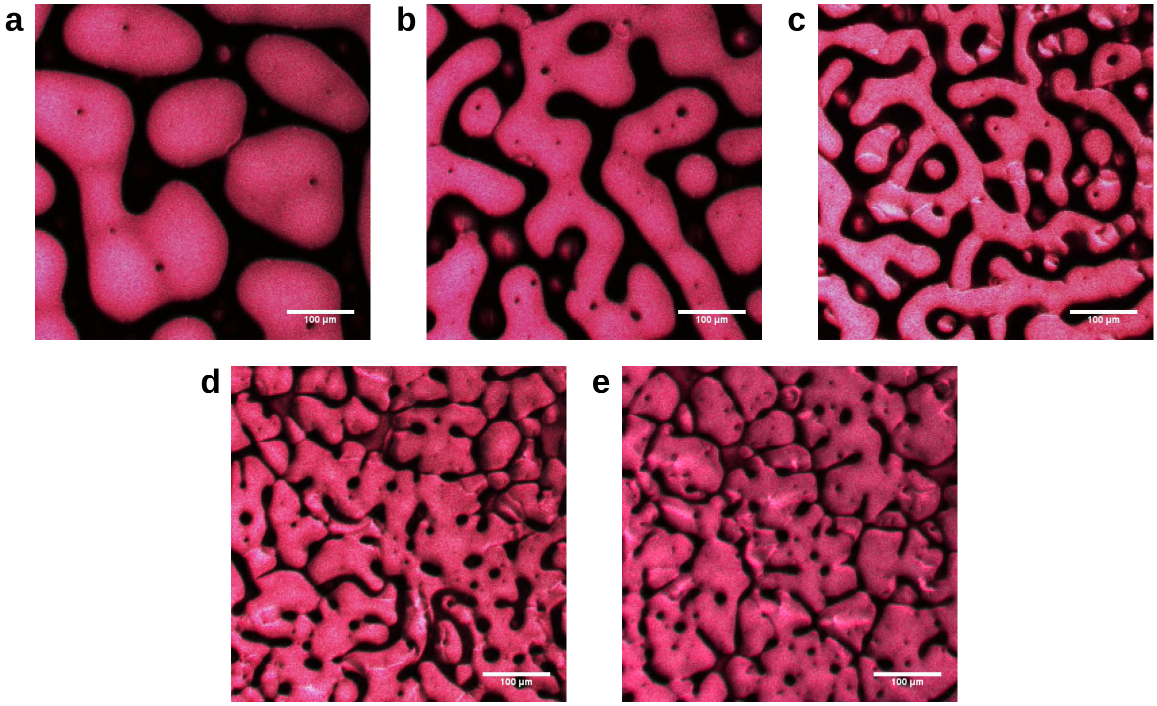

Supplementary Figure S18 | Effect of cooling rate on characteristic length scale of initial phase decomposition. Bright field images of the AuNP-LC composite during the phase transition when cooled at a rate of **a** 0.1°C/min, **b** 0.5°C/min, **c** 1.0°C/min, **d** 5.0°C/min, **e** 10.0°C/min, and **f** 20.0°C/min. The scale bar represents 100 µm .

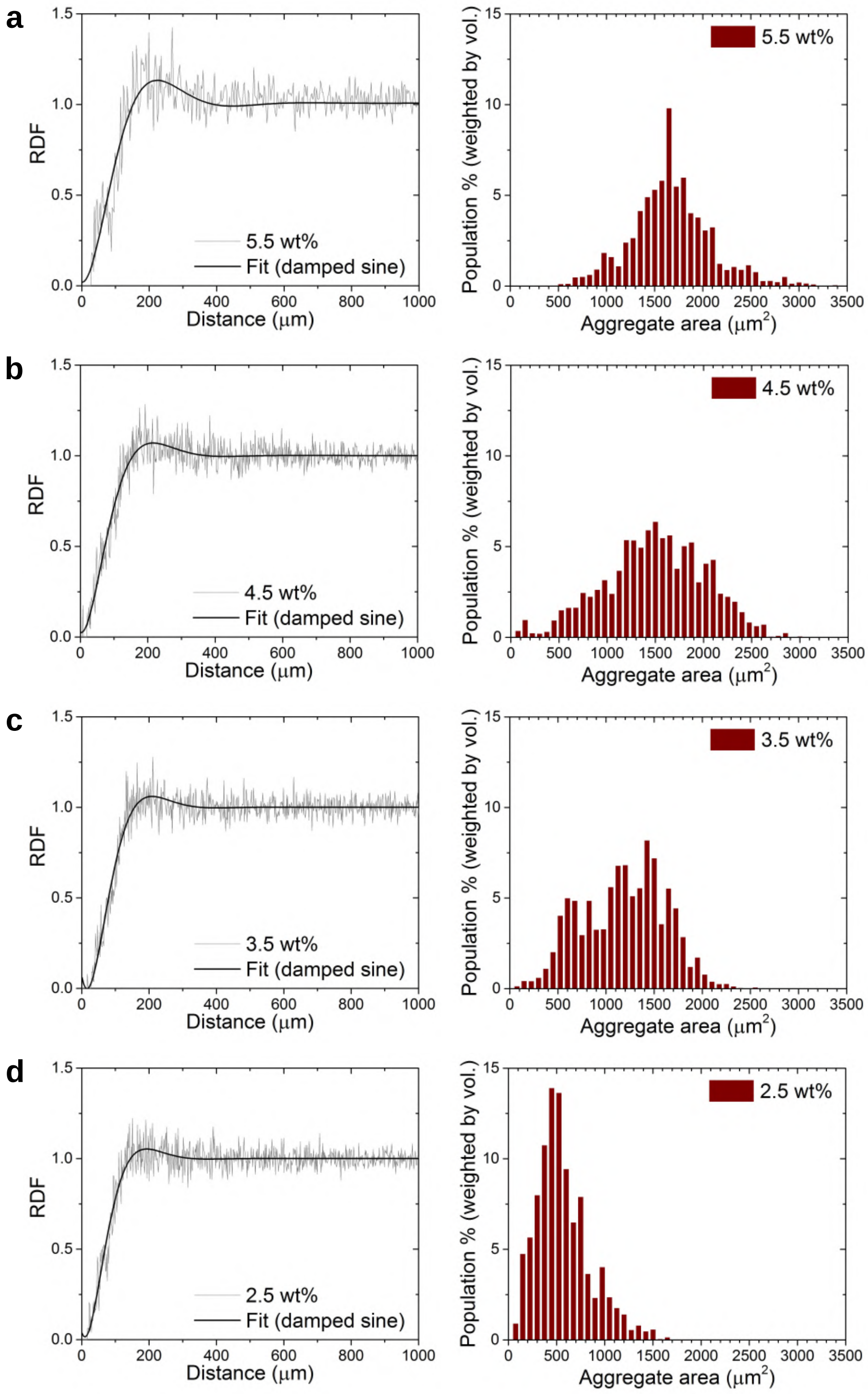

Supplementary Figure S19 | Effect of AuNP concentration. AuNP-LC composites containing **a** 5.5wt%, **b** 4.5wt%, **c** 3.5wt%, and **d** 2.5wt% of AuNPs in 5CB were each cooled at 0.5°C/min. Bright field images were used to obtain aggregate histograms and the RDF. As the concentration decreased, the mean aggregate size also decreased. Similarly, there was a decrease in the magnitude of the peak in the RDF that described the characteristic spacing. This indicates that lower concentrations have a lower degree of ordering. As the temperature is reduced, the initial phase decomposition leads to NP-enriched isotropic and NP-depleted nematic regions. At higher concentrations, the isotropic regions lose some AuNPs by diffusion into the nematic phase but enough remain to form an aggregate. At lower concentrations, however, some of these regions may be below a critical local concentration to allow for an aggregate to remain, thus leading to a decrease in the spatial order due to the partial loss of nearest neighbour aggregates.

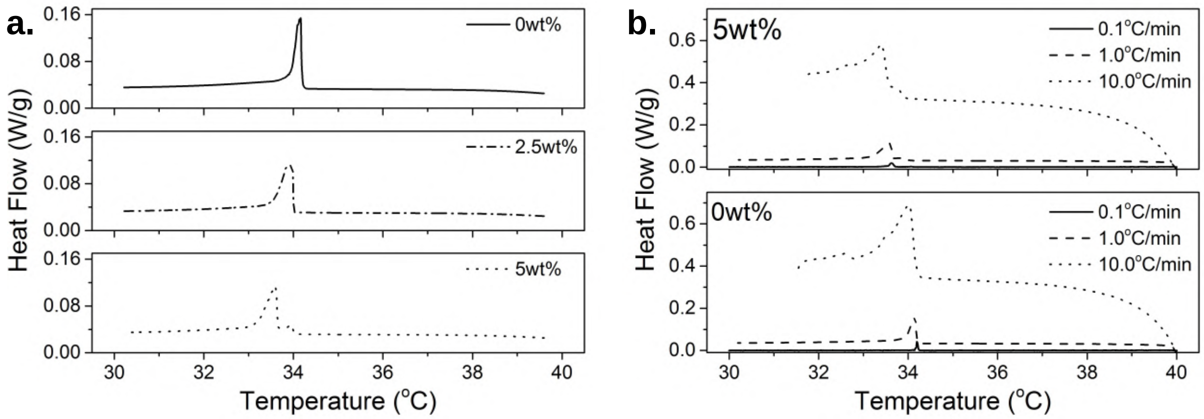

Supplementary Figure S20 | DSC analysis of 5CB and AuNP-LC composite. **a**. The phase transition temperature of pure 5CB and AuNP-LC composites using DSC. **a**. The effect of concentration was investigated by varying the concentration of the AuNPs in the LC. **b**. The effect of cooling rate was investigated for pure 5CB and 5wt% of AuNPs in 5CB.

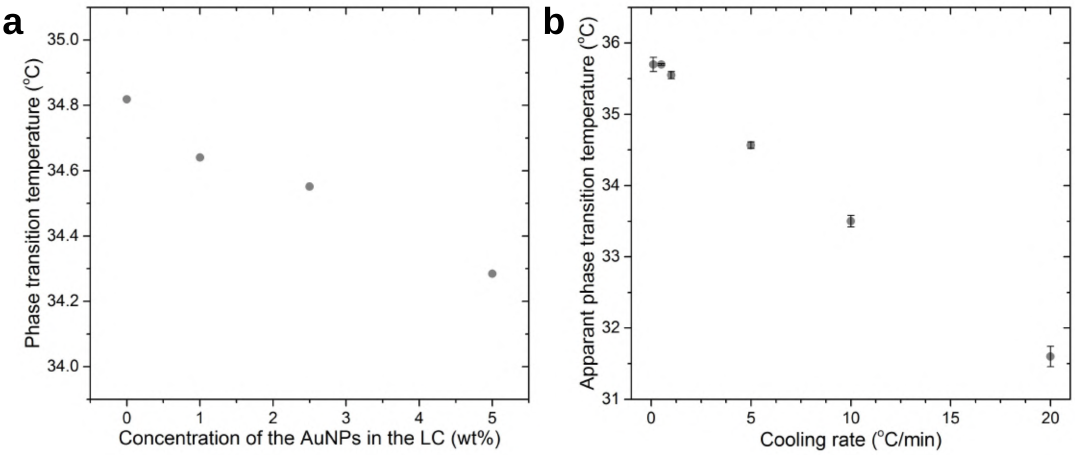

Supplementary Figure S21 | Phase transition temperature of the AuNP-LC composite. **a**. The phase transition temperature as a function of concentration was investigated. AuNP-LC composites with varying concentrations of AuNPs were prepared and placed on a microscope slide. The composites were then cooled at 0.5°C/min and the phase transition temperature recorded. **b**. The effect of cooling rate on the phase transition temperature was also determined for a pure 5CB sample. Error bars are included for each data point. Note: the absolute temperature reading was calibrated using a reference cell filled with pure 5CB. The reference cell comprised of a glass sandwich and a thermocouple recorded the temperature of the centre of the cell.

The RDF represents the average spacing of aggregates over the full radial profile without directional information. To evaluate anisotropy, the positions of the aggregates were plotted in polar coordinates. These values were then presented as a 2D histogram for clarity. In order to evaluate the experimental results shown in Figure 6 (main text) and in Figure 22, a regular hexagonal pattern was investigated with increasing Gaussian noise in Figure 23.

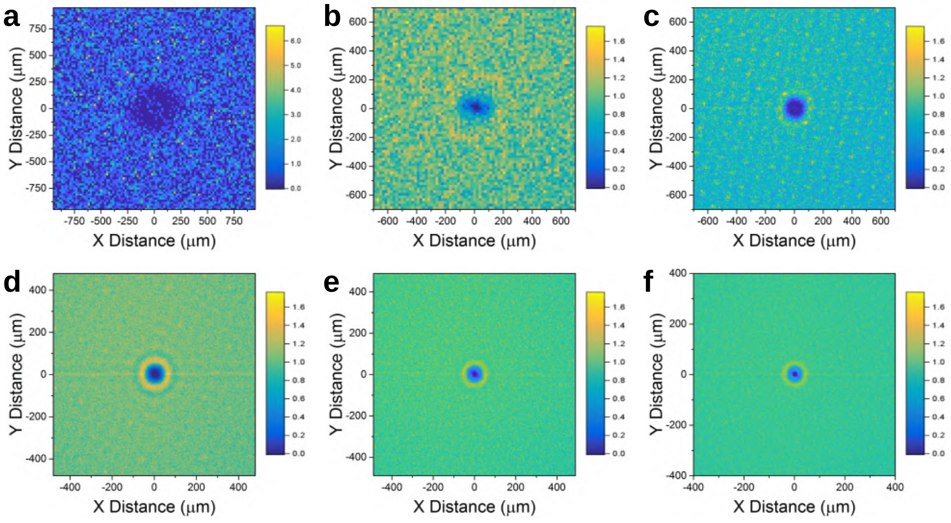

Supplementary Figure S22 | 2D alignment of AuNP aggregates in the LC - effect of cooling rate (experimental). The AuNP-LC composites were cooled at **a** 0.1°C/min, **b** 0.5°C/min, **c** 1°C/min, **d** 5°C/min, **e** 10°C/min, and **f** 20°C/min. The relative positions between each aggregates were determined and the results were normalised and displayed in a 2D histogram.

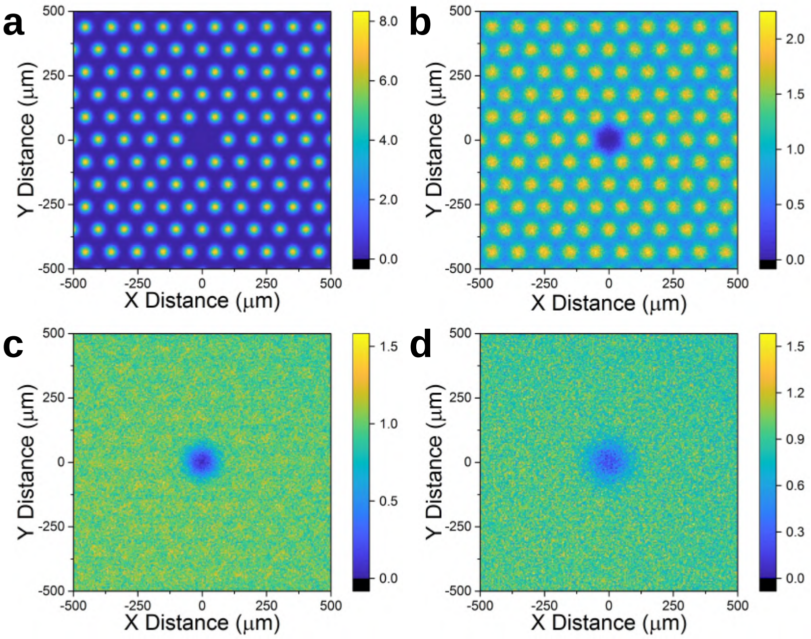

Supplementary Figure S23 | 2D alignment of AuNP aggregates in the LC - effect of disorder (simulation). A hexagonal array was created with added gaussian noise with a standard deviation of **a** 0.1, **b** 0.2, **c** 0.3, and **d** 0.4. The relative positions between each point were determined and the results were normalised and displayed in a 2D histogram.

References

1. Martyna, G. J., Tobias, D. J. & Klein, M. L. Constant pressure molecular dynamics algorithms. *J. Chem. Phys.* **101**, 4177 (1994).
2. Anderson, J. A., Lorenz, C. D. & Travesset, A. General purpose molecular dynamics simulations fully implemented on graphics processing units. *J. Comput. Phys.* **227**, 5342 (2008).
3. Glaser, J. *et al.* Strong scaling of general-purpose molecular dynamics simulations on GPUs. *Comput. Phys. Commun.* **192**, 97 (2015).
4. Frenkel, D. & Smit, B. *Understanding Molecular Simulation* (Academic Press, 2002).
5. Boettinger, W., Warren, J., Beckermann, C. & Karma, A. Phase-field simulation of solidification. *Annu. Rev. Mater. Res.* **32**, 163–194 (2002).
6. Milette, J., Toader, V., Reven, L. & Lennox, R. B. Tuning the miscibility of gold nanoparticles dispersed in liquid crystals via the thiol-for-DMAP reaction. *J. Mater. Chem.* **21**, 9043–9050 (2011).
7. Badia, A. *et al.* Structure and chain dynamics of alkanethiol-capped gold colloids. *Langmuir* **12**, 1262–1269 (1996).
